# Trophic ecology of the African riverine elephant fishes (Mormyridae)

**DOI:** 10.1002/ece3.70173

**Published:** 2024-08-27

**Authors:** Gina Maria Sommer, Samuel Didier Njom, Adrian Indermaur, Arnold Roger Bitja Nyom, Kateřina Jandová, Jaroslav Kukla, Miloslav Petrtýl, Petra Horká, Zuzana Musilova

**Affiliations:** ^1^ Department of Zoology, Faculty of Science Charles University Prague Czech Republic; ^2^ Department of Biological Sciences University of Ngaoundéré Ngaoundéré Cameroon; ^3^ Zoological Institute University of Basel Basel Switzerland; ^4^ Department of Management of Fisheries and Aquatic Ecosystems University of Douala Douala Cameroon; ^5^ Institute for Environmental Studies, Faculty of Science Charles University Prague Czech Republic; ^6^ Department of Zoology and Fisheries, Faculty of Agrobiology, Food and Natural Resources Czech University of Life Sciences Prague Suchdol Czech Republic

**Keywords:** mormyrids, stable isotopes, trophic ecology, trophic position, trophic resource portioning

## Abstract

Multiple species of the elephant fishes (Mormyridae) commonly coexist in sympatry in most African tropical rivers and lakes. In this study, we investigated the trophic ecology and potential trophic niche partitioning of eleven mormyrid fish species from the Sanaga River system in Cameroon using the stable isotope composition of carbon and nitrogen in the muscle samples. Albeit most mormyrids mainly feed on invertebrates, we found differences in isotope ratios, and we report signs of the trophic niche partitioning among species. We further found significant differences in isotopic signatures within the *Mormyrus* genus, suggesting ecological niche diversification among three closely related species. We have also evaluated differences in the isotopic signals between seasons in four species, which could be possibly caused by species migration and/or anthropogenic agricultural activities. To evaluate body shape, we applied geometric morphometric analyses, and we show that most of the species are clearly morphologically separated. We focused on the mormyrid ecomorphology to identify a possible interaction between shape and ecology, and we found a relationship between the δ^13^C (but not δ^15^N) isotopic signal and morphology, suggesting their interplay during mormyrid evolution. Overall, we present robust evidence of the trophic niche partitioning within the mormyrid species community, and we integrate trophic ecology with morphometrics, shedding light on the enigmatic evolutionary history of these fascinating African fishes.

## Graphical abstract

The study is focused on the trophic ecology of Mormyridae based on the stable isotope analysis of carbon and nitrogen. We report on differences of the isotopic signal among eleven mormyrid species from the Sanaga River in Cameroon, and we provide evidence for trophic niche differentiation within the genus Mormyrus. We also noticed a potential relationship between the snout shape and the trophic position, and we further found an effect of seasonality on isotopic signatures.

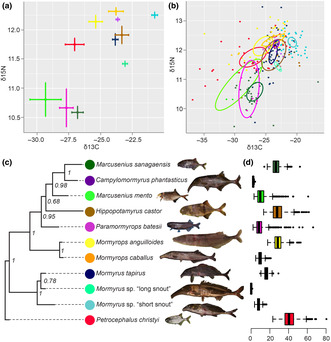

## 1 INTRODUCTION

Elephant fishes, Mormyridae, exclusively occur in the freshwater habitats of Africa and have diversified into more than 230 species, the most of all osteoglossomorpha families (Fricke et al., 2023). The mormyrid evolutionary success is often attributed to their novel electric sensory system used for orientation/electrolocation and conspecific communication (Carlson et al., 2011; Sullivan et al., 2002). Both natural and sexual selection on the electric signals seem to have contributed to speciation in these fish (Arnegard et al., 2010). Thanks to their interesting biology, enormous brains, and the additional sensory system, mormyrids often serve as model species for neurobiology, neuroethology, behavioral ecology, and physiology, yet surprisingly little is known about their general life history.

Ecological studies on habitat preference, reproductive behavior, or feeding strategies are scarce in mormyrids but have revealed complex behavioral traits, such as pack hunting (*Mormyrops anguilloides* in Lake Malawi; Arnegard & Carlson, 2005) or courtship sound production (*Pollimyrus isidori*; Crawford et al., 1997). Substantial seasonal migrations have been reported for mormyrids in several river systems (Blake, 1977; Okedi, 1965; Van der Waal, 1996). These migrations are putatively associated with shifts in diet and a trophic position (e.g., McMeans et al., 2019; Nashima et al., 2020).

Studies on trophic ecology show that mormyrids are mainly invertivorous (Adjibade et al., 2019; Peel et al., 2019; Winemiller & Adite, 1997) or rarely piscivorous (*Mormyrops anguilloides*; Bailey, 1994; Skelton, 2001). Differences in trophic preferences have previously been described in coexistent species from Lake Ayame, Ivory Coast (Kouamélan et al., 2006) or Sokoto‐Rima River basin, Nigeria (Hyslop, 1986) based on the stomach content analyses suggesting that different species may specialize on different groups of insect larvae. However, comparative studies focused on the trophic ecology at the community level are lacking for mormyrids. The stable isotope analysis by mass spectrometry is a powerful tool to study trophic ecology. Heavier stable isotopes of carbon (^13^C), nitrogen (^15^N), or sulfur (commonly ^34^S) accumulate differently in the organisms compared to the common isotope since the lighter isotopes form weaker bonds than the heavier ones (jardine et al., 2003). The isotopic signal revealed from muscle tissue reflects much longer time span of feeding (weeks/months) compared to just a few hours/days if examining the stomach content. Therefore, the isotopic signature can be used to study flow of energy through a food web (Joni et al., 2015; Qin et al., 2021), to demonstrate trophic specializations or niche differentiations in populations (Stewart et al., 2021) and closely related species (Wang et al., 2022), or, lastly, to detect seasonal diet differences (Andrade et al., 2023).

The ecological niche of a species is too complex to be measured as a whole. By focusing on the trophic ecology, the stable isotope ratios can be used to assess the so‐called isotopic niche, which is defined as the area in δ space with isotopic values as coordinates (Newsome et al., 2007). While the δ^15^N values changes are mainly correlated with a trophic level, the δ^13^C changes are connected with a variable source of primary C. Such calculated isotopic niches can then be statistically compared, namely, as size and overlap of isotopic niches among different species (Swanson et al., 2015). Reports based on stable isotope analyses in mormyrids are scarce (Arnegard et al., 2010; Soto et al., 2019) while being more commonly used in other African fishes (e.g., the adaptive radiations of cichlids, Muschick et al., 2012).

Ecomorphology studies interactions between morphology and ecology (Bower & Piller, 2015; Curran, 2015; Evans et al., 2019; Sibbing et al., 1994), and the elephant fishes as a diverse fish family are ideal candidates for such questions (Feulner et al., 2007). Mormyrids contain 22 genera with remarkable variation in their snout shape. It ranges from short and completely rounded snouts (e.g., in *Brevimyrus* and all Petrocephalinae), through more elongated lower lips (*Marcusenius* and *Hippopotamyrus*) to elongated snouts (*Mormyrops*), long protrusions (*Mormyrus*), or an extreme downward‐shaped rostrum (*Campylomormyrus*). The fact that different mouth shapes often facilitate feeding on different resources (prey/habitats) is notoriously known for bird beak morphologies (e.g., in Darwin finches (Reaney et al., 2020) or waterfowls (Olsen, 2017)) but was also reported for cichlid fishes (Soria‐Barreto et al., 2019; Tada et al., 2017). Very few studies focused also on ecomorphology in the elephant fishes. Feulner et al. (2007, 2008) investigated diversification in *Campylomormyrus* and found that shape of the snout protrusions may be associated with different trophic specializations, although this needs further confirmation.

The main objective of this study was to compare the trophic ecology of eleven species of mormyrids from the Sanaga River (Cameroon) and to provide an insight into their community ecology using stable isotope analysis. First, we expected species inhabiting the same habitat to depict contrasting trophic niche favoring species co‐existence. We focused on the overall community signal (all species and genera), as well as we gave additional attention to pairwise comparison of closely related species (multiple species within three genera). Second, we investigated patterns of trophic signatures of fish and resources over different seasons to assess the role of seasonal fish migration and/or changes in food resource in modulating mormyrid trophic ecology. Finally, we explored associations between morphological traits and trophic preferences of fish to reveal the potential existence of ecomorphological patterns in mormyrids. Specifically, we aimed to test if (head) morphology was associated anyhow with the trophic preferences and, as such, provide a motivation for further ecomorphological studies on mormyrid snouts. Finally, we performed a phylogenetic analysis of the studied species to confirm the phylogenetic position of two undescribed *Mormyrus* species and to complement our results with an evolutionary perspective. Overall, our study contributes to a better understanding of the Cameroonian freshwater fish diversity, biology, and ecology in one of the less regulated rivers in Africa (until recently) and highlights the need for further ecological studies on mormyrids.

## 2 MATERIALS AND METHODS

### 2.1 Sampling

**FIGURE 1:**
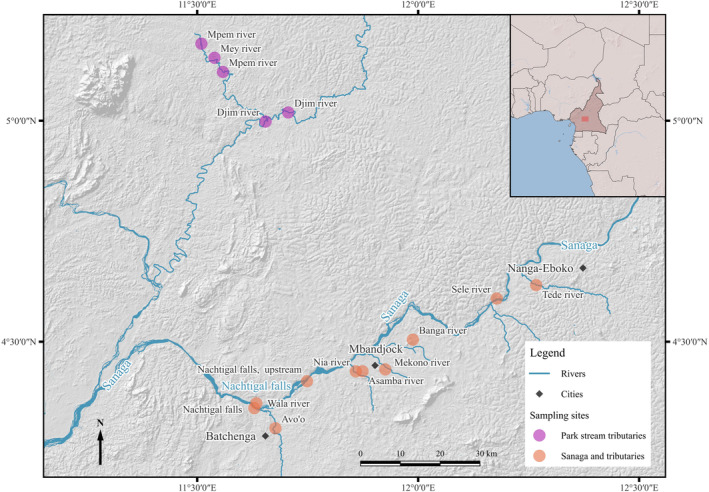
Overview of the study site: Map of the section of the middle Sanaga river close to the Nachtigal Falls (Chutes de Nachtigal) in Cameroon, Africa. The most frequented sampled sites are up‐ and downstream of Nachtigal falls and are in the vicinity of the town Batchenga (black dot). (Basemap: https://Www.esri.com/en‐us/maps‐we‐love/overview, borders: https://www.divA‐gis.org/gdata, rivers, streams and places: openstreetmap.orG).

**FIGURE 2:**
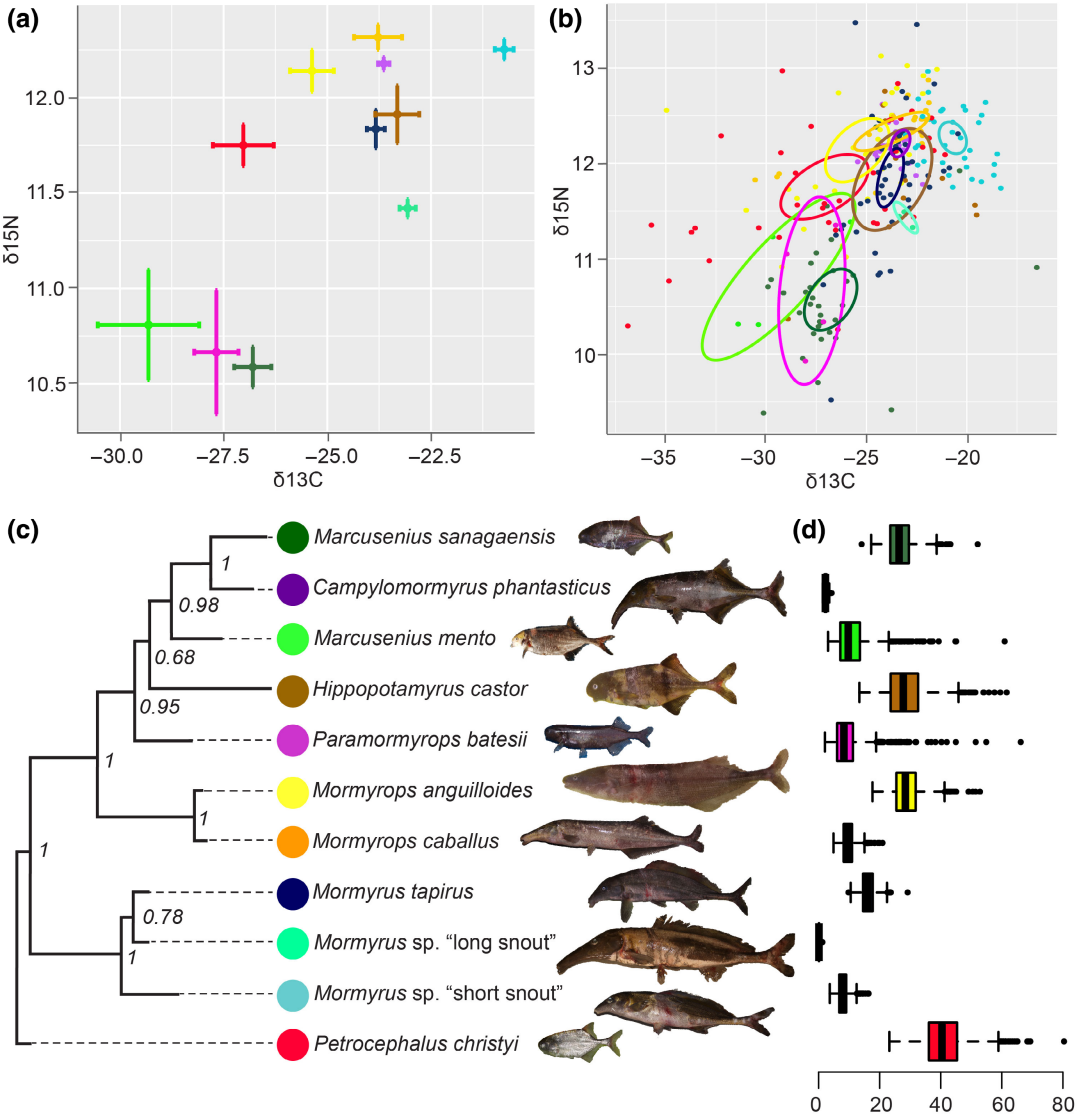
(a) Stable isotope values (δ^13^C and δ^15^N; ‰) of eleven mormyrid species sampled in the Sanaga River. Horizontal and vertical error bars represent standard error around the mean. (b) SIBER ellipses of each mormyrid species. Dots display individuals and 99% confidence ellipses are represented. Note that the plots do not include the transition season samples specifically addressed in the text and in Figure 5. Note the different scale of plot (a) and (b). (c) Phylogenetic relationships of the studied mormyrid species based on the COI sequences and reconstructed by Bayesian inference. (d) Box plots of the isotopic niche sizes calculated using the nicheROVER package (*α* = .95 and 1000 Monte Carlo draws). Note that species colors are shown in panel (c).

We sampled mormyrid fishes in Cameroon in 2017–2018. The study sites were at the Sanaga River in Cameroon, the country's longest river (918 km; Dubreuil et al., 1975). The Sanaga River has three large contiguous segments, Upper Sanaga, Middle Sanaga, and Lower Sanaga (Bitja Nyom et al., 2020), and this study took place at Middle Sanaga. We sampled at two main locations: (1) in the proximity of the cascades of Nachtigal (up‐ and downstream), as well as at eight Sanaga tributaries (orange dots in Figure 1) and (2) at five localities of the Park stream, an inflow stream of the Sanaga River (purple dots in Figure 1). Sanaga is located in the Guinean equatorial climate zone (Amougou et al., 2015; Olivry, 1986), and we sampled in different seasons here, namely, a main wet season (from August until November) and a main dry season (from December to March), plus a transition season between the dry and the wet season (from April until July), sometimes also divided as a shorter wet season lasting from April until June and a shorter dry season (from July until early August; Bitja Nyom & Pariselle, 2015).

The fish were continuously sampled in 2017 and 2018 but the number of individuals per species varied and the representation of each season was not always complete and at times unequally distributed (see Table 1). The individuals were caught using multiple gillnets of different mesh sizes (varied from 1 to 5 cm) and processed shortly upon capture close to the collection sites. After euthanizing the fish with a lethal amount of MS‐222, photographs and measurements were taken, and fin clips were collected for DNA analyses. Afterwards, a 1–2 cm piece of lateral muscle was dissected from an area between the ventral and the caudal fin in proximity to the lateral line and fixed in 96% ethanol.

**TABLE 1:** Overview of collected species and their representation.

Species	Total	Sanaga River and tributaries					Park stream and tributaries				
		D 2017	W 2017	D 2018	T 2018	W 2018	D 2017	W 2017	D 2018	T 2018	W 2018
*Campylomormyrus phantasticus*	32	5		16	4	7					
*Hippopotamyrus castor*	18	3	1	6	1	4		1	1	1	
*Marcusenius mento*	4							1		3	
*Marcusenius sanagaensis*	43	2	11	8	7	4		3	5	3	
*Mormyrops anguilloides*	31	9	4	5	1	12					
*Mormyrops caballus*	21	2	1	14		4					
*Mormyrus tapirus*	53	15	2	18	2	12				4	
*Mormyrus* sp. “long snout”	3	3									
*Mormyrus* sp. “short snout”	35	8	3	21		3					
*Paramormyrops batesii*	4							1	3		
*Petrocephalus christyi*	44	4		23	8	1			8		

*Note*: There are no transition samples from 2017; D stands for dry season, T for transition and W for wet season. Please note that *Mormyrus* sp. “short snout” and *Mormyrus* sp. “long snout” refer to so far undescribed species and thus have these preliminary names in this study.

In total, we collected 288 individuals of eleven species (*Campylomormyrus phantasticus* (Pellegrin, 1927), *Hippopotamyrus castor* Pappenheim, 1906, *Marcusenius mento* (Boulenger, 1890), *Marcusenius sanagaensis* Boden et al., 1997, *Mormyrops anguilloides* (Linnaeus, 1758), *Mormyrops caballus* Pellegrin, 1927, *Mormyrus tapirus* Pappenheim, 1905, *Mormyrus* sp. “short snout,” *Mormyrus* sp. “long snout,” *Paramormyrops batesii* (Boulenger, 1906), and *Petrocephalus christyi* Boulenger, 1920). All fish were collected with appropriate research permits, namely, N° 007, 048, 049/MINRESI/B00/C00/C10/C12 and N° 2376/PRBS/MINFOF/SETAT/SG/DAFP/SDVEF/SC/ENJ granted by the Ministry of Scientific Research and Innovation and the Ministry of Forestry and Wildlife in Cameroon.

All samples were fixed in 96% ethanol in the field and stored in a freezer at −20°C later at the laboratory facility. Prey samples of mormyrids were collected by dissecting the stomach contents of nine individuals (two *M. caballus*, one *M. anguilloides*, two *H. castor*, two *M. tapirus*, and one *M*. sp. “short snout”) as well as by capturing benthic samples, that is, plant material and invertebrates. The latter were collected from the river bed area, the riverbank, and the water column of the Sanaga River at the Nachtigal falls and its tributary Wala River. The organisms from these environmental samples were identified taxonomically under a stereomicroscope to the highest level possible, from a class (Gastropoda), mostly to an order (e.g., Ephemeroptera), and some samples to the family level (e.g., Hydrophilidae, Elmidae) with the help of pictures taken from stomach content material and invertebrate identification guides (Mary, 2017; Moisan et al., 2010). The water plant material was not further identified, and the various plants were mixed to achieve an amount of at least over 2 mg (as ideally would be 3 mg) and labeled accordingly as the group called “plant.” The isotope values generated by both the stomach content material and the benthic material are jointly represented when later referring to the values of the “prey samples,” unless otherwise specified.

### 2.2 Sample processing

The muscle samples were oven‐dried at 60°C for 48 h or at 45°C for 72 h. Any bone structures were carefully removed with a clean forceps if present. The samples were homogenized using a ball mill (MM400, Retsch, Germany) and a sample aliquot of 0.50 mg (±0.05 mg) was folded into a tin capsule. The prey samples were processed in the same manner, except that grinding was performed partly manually using pestle and mortar. Some individual prey samples were pooled together to provide enough material for stable isotope analyses (i.e., Plecoptera, Trichoptera, and Ephemeroptera). Total carbon and nitrogen content as well as their isotopic ratios δ^13^C and δ^15^N were measured by a Delta V Advantage mass spectrometer coupled to a Conflo IV and an elemental analyzer Flash 2000 (all instrumentation by Thermo Fisher Scientific, Bremen, Germany). The carbon and nitrogen isotope ratios are expressed as delta notations, which is the following: δX = 1000*(Rsample/Rstandard – 1), where X stands for ^13^C or ^15^N, respectively, and R is the carbon or nitrogen isotope ratio (R = ^13^C/^12^C or ^15^N/^14^N). Repeated measurements of a series of international standards (IAEA‐CH3, IAEA‐CH6, IAEA‐600, IAEA‐N1, IAEA‐N2, IAEA‐NO3) were applied to normalize the measured isotope ratios to the Vienna Pee Dee Belemnite (VPDB) and atmospheric N2 scales (coplen, 1996). In addition, a glycine standard was measured after every 10th sample to provide calibration for elemental composition and a quality control for isotopic measurement. Analytical precision was within ±0.2 ‰ for both δ^13^C and δ^15^N.

### 2.3 Stable isotope analyses

#### 2.3.1 Biplots and niche overlaps

The δ^13^C and δ^15^N values were analyzed in R Studio Statistical Software [version 1.4.1717; RStudio Team, 2021, http://www.rstudio.com/] using the R package SIBER (Jackson et al., 2011) to visualize the data and plot the ellipses in Figure 2b. Furthermore, the probabilistic method within the R package nicheROVER (Swanson et al., 2015) was utilized to calculate the niche overlaps (i.e., the pairwise probabilities of one species being included in the trophic niche of another species) as well as niche sizes with *α* = 0.95 (α presents the overlap metric at two niche regions sizes for comparison probability of overlap, authors suggest 95% as default), which gives a value of the hypervolume of the niche size.

#### 2.3.2 Further statistical tests

We tested for differences in isotopic signatures between closely related species. We pairwise compared different species of the same genus, for which we performed *t*‐tests with unequal variances in Microsoft Excel® version 2013 individually for the δ^13^C values and the δ^15^N values of three *Mormyrus species*, two *Mormyrops*, and two *Marcusenius* species. The alpha‐value for the multiple *Mormyrus* genus comparisons was Bonferroni‐adjusted. The effect of season was also assessed in Microsoft Excel with separately run one‐way ANOVAs for each species and each stable isotope. Afterwards Tukey HSD tests, which adjust for multiple comparisons were performed in R to allow pairwise comparison among the seasons. In case normality was violated, the nonparametric tests Mann–Whitney‐*U* for the interspecies pairwise comparisons (i.e., the δ^13^C data for *Marcusenius sanagaensis, Mormyrops anguilloides* and *M. caballus*) and Kruskal–Wallis tests with Dunn's test for the multiple testing for season (the δ^13^C data for *Marcusenius sanagaensis* from the dry and transition season, *Campylomormyrus phantasticus*—wet season and *P. christyi*—transition season) were performed instead (also in R).

#### 2.3.3 Trophic position calculations

The R package “tRophicPosition” (Quezada‐Romegialli et al., 2018) was used to assess the trophic position of the studied species using one baseline of all benthic samples from the prey samples. We also performed the supplementary analysis with a baseline from the stomach content‐derived prey samples for comparison (see Figure S3). As no specific trophic discrimination factors (TDF) have been studied before for mormyrids, the established TDF of McCutchan (McCutchan et al., 2003) were used for a fish muscle tissue, which means a mean of 2.9‰ ± 0.32 SE for δ^15^N and 1.3‰ ± 0.3 SE of for δ^13^C. The trophic position (TP) was estimated using a trophic level (λ) of 2 with primary consumers as a baseline. The conventional range of trophic levels ranges from 1 (primary producers) to 5 (2–4.5 for fishes in freshwater ecosystems; Trites, 2001; Yoğurtçuoğlu et al., 2020). The incorporated Bayesian model of the package “tRophic Position” was run with 4 chains and for 20,000 iterations.

### 2.4 Molecular phylogeny of the mormyrid species

We reconstructed the phylogenetic relationships of the Sanaga mormyrid community. We used the mitochondrial CO1 sequence of 11 species from 7 genera co‐occurring at the study locations in the Sanaga River. The collected fin clips of ten species were used for DNA‐extractions using the DNeasy Blood and Tissue kit (Qiagen) following the manufacturer's protocol. The mitochondrial marker cytochrome oxidase subunit I (COI) was amplified via PCR with universal barcoding fish primers (forward—FishF1–5′‐TCA ACC AAC CAC AAA GAC ATT GGC AC‐3′ and reverse FishR1–5′‐TAG ACT TCT GGG TGG CCA AAG AAT CA‐3′ from Ward et al., 2005). PCR conditions were 94°C for 4 min; 35 cycles of 45 s at 94°C; 45 s at 52°C; and 45 s at 72°C; final elongation for 5 min at 72°C and holding at 10°C. PCR products were checked visually by electrophoresis on a 1.5% agarose gel. PCR product purification happened with ExoSAP‐IT®. Sequencing was conducted by Macrogen Europe. The sequences are available on GenBank under the accession numbers: PP776773‐PP776792. In addition, we added the COI gene for *Marcusenius mento* from the GenBank sequence (acc. no. HM882757 and HM88272) as fin clips were not available for this species. Bayesian inference (BI) was performed in MrBayes version 3.2.6 (Ronquist et al., 2012) by Metropolis‐coupled Markov chain Monte Carlo (MC3) sampling for 1 million generations. The chain was sampled every 100th generation and the first 25% were discarded as burn‐in. The used evolutionary rate model was the GTR substitution model with gamma‐distributed rate variation across sites and a proportion of invariable sites. Two sequences of each species were used to reconstruct the phylogenetic tree and *Petrocephalus christyi* was used as outgroup as the subfamily Petrocephalinae (all *Petrocephalus* species) are the confirmed sister group to all other Mormyridae species (subfamily Mormyrinae) by many previous phylogenetic studies (e.g., Lavoué et al., 2000, 2012; Taverne, 1969, 1972).

### 2.5 Geometric morphometric analysis

We performed morphometric analysis on nine out of 11 recognized taxa. Two species had to be excluded due to the insufficient number of individuals with a photo. All used specimens (*n* = 155) were photographed on the left body side with a scale bar. Landmark‐based geometric morphometric methods were used to record coordinates of 13 homologous landmarks and capture information of body shape using TpsDig (Rohlf, 2003). Landmark configuration on the mormyrid body (Figure 6a) was modified following Feulner et al. (2007) and Arnegard et al. (2010). All the following morphometric analyses were conducted in the MorphoJ software (ver. 1.08.01; Klingenberg, 2011). First, differences due to size, orientation, and position were removed by generalized Procrustes analysis (Rohlf, 1999; Slice, 2001). After superimposition, the data were converted into principal warps using the thin plate spline model (Bookstein, 1989). These variables can then be used in conventional multivariate analyses because they possess the same number of variables as degrees of freedom (Zeldith et al., 2004). We performed the principal component analysis (PCA) of morphological variables to demonstrate the shape differences among studied taxa. We further applied Two‐block Partial Least Squares (PLS) analyses as implemented in the MorphoJ software to examine the relationship between the shape (all Procrustes coordinates combined, plus the first three PCA axes separately) and δ^13^C and δ^15^N isotopic signals to demonstrate possible association of morphology with trophic ecology. PLS analysis is derived from a multivariate correlation technique that describes the covariation between two sets of variables (see, e.g., Rohlf & Corti, 2000). The significance of this correlation is assessed using a randomization test. PLS technique models the covariation between two sets of variables, here shape and isotope variables, as a product of linear combinations of each of the two sets of variables. The linear combinations are computed by using a singular‐value decomposition of the matrix of covariances between the shape variables and the isotope variables, and the linear combinations are constructed to maximize the covariation between the two sets of variables. Our geometric morphometric analyses and the subsequent PLS analyses were performed following the application as in Adams and Collyer (2016), Haines et al. (2023), and McGuire and Lauer (2020). In total, eight separate models were run, four for the δ^13^C values and four for the δ^15^N values (against combined Procrustes coordinates and three PCs separately). PLS were tested for significance using a permutation test (based on 1000 random permutations).

## 3 RESULTS

### 3.1 Phylogenetic relationships among mormyrid species

In our phylogeny, we resolved all genera with multiple species to be monophyletic, except for *Marcusenius*, as *Campylomormyrus phantasticus* clustered within the *Marcusenius* genus (Figure 2c). We further confirmed that two undescribed species, *Mormyrus* sp. “short snout” and *Mormyrus* sp. “long snout” belong to the genus *Mormyrus* but are different from the described species *Mormyrus tapirus*. Furthermore, there does not seem to be an obvious phylogenetic signal in the observed trophic results (comparing Figure 2a,c), albeit we did not specifically test for this.

### 3.2 Differential isotopic signals in multiple species and genera

**FIGURE 3:**
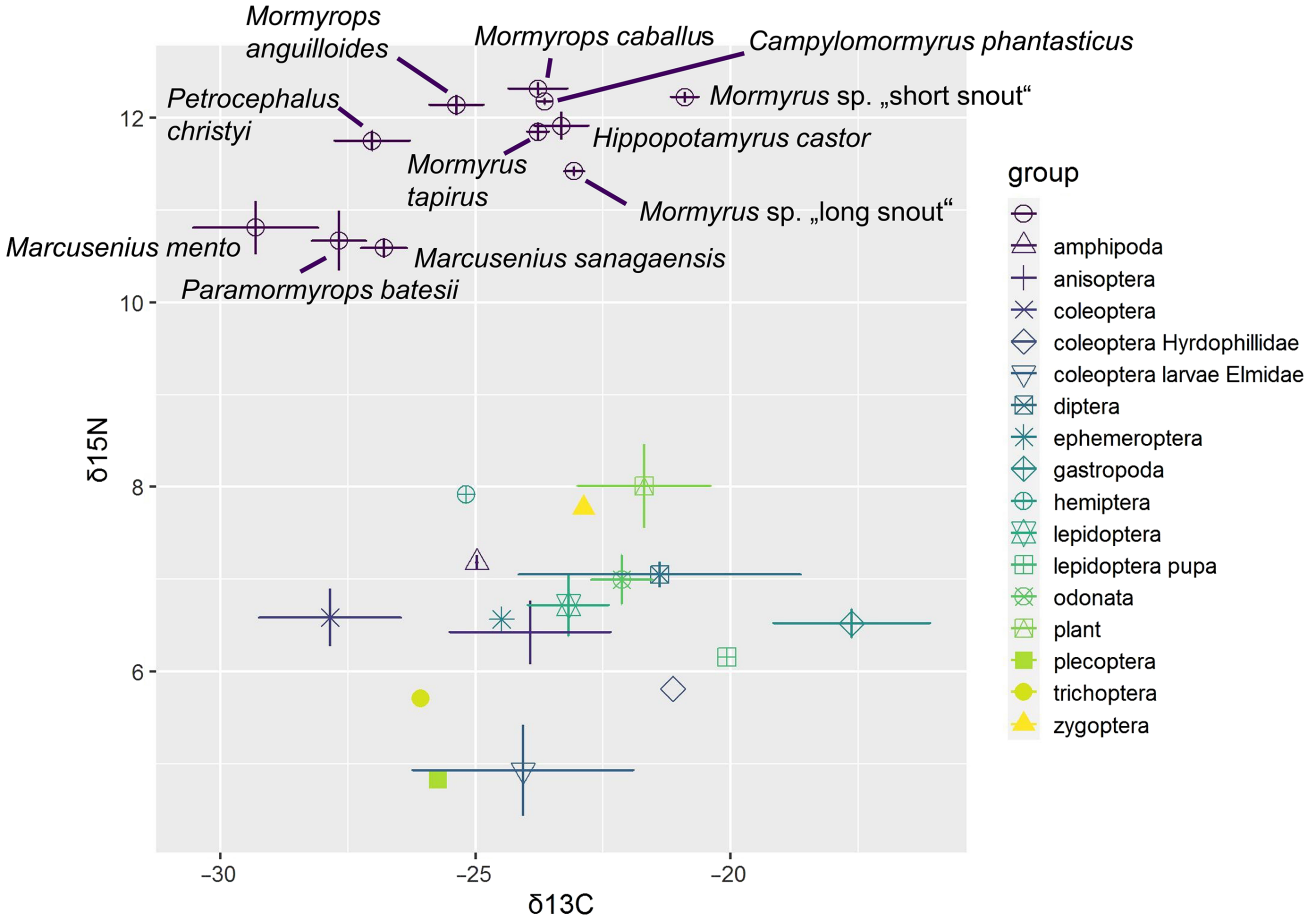
Isotopic bi‐plot (δ^13^C and δ^15^N; ‰) of mormyrid muscle tissue (purple dots) and putative prey. Horizontal and vertical error bars represent standard error around the mean.

Based on 258 samples of 11 species of the mormyrid community in the Sanaga River, we found that the stable isotope signals partly differed among the species (Figure 2; see Table S1 for raw values). The niche sizes also differed among species (Figure 2d) and we found substantial niche overlaps for some species, while low probability of overlap with another species combinations (Table 4). The two *Marcusenius* species and *Paramormyrops batesii* shared the lowest δ^13^C and δ^15^N values in comparison to all other species (Figure 2). Interestingly, certain species from different genera possessed similar isotopic signatures, such as *Hippopotamyrus castor* and *Mormyrus tapirus*, or *Mormyrops caballus* and *Campylomormyrus phantasticus*, whereas the species from the same genus were more separated from each other. This was especially obvious in the *Mormyrus* genus (Figures 2, 3, 6f, Table 4). The niches differed in size among species (Figure 2d), with some species having just a small and narrow niche (e.g., *Campylomormyrus phantasticus*; niche size = 2.29), while other species had higher variance and a bigger niche size (e.g., *Petrocephalus christyi*; niche size = 43.95).

### 3.3 Prey samples

Sixteen potential prey groups were determined: Amphipoda, Anisoptera, Coleoptera, Coleoptera Hydrophillidae, coleopteran larvae Elmidae, Diptera, Ephemeroptera, Gastropoda, Hemiptera, Lepidoptera, Lepidoptera pupa, Odonata, plant, Plecoptera, Trichoptera, and Zygoptera. Only three groups (Anisoptera, Gastropoda, and Elmidae (Coleoptera)) were simultaneously present in the stomach samples and the benthic items (see Figure S1 and Figure S2). As expected, mormyrid fish muscles had a higher trophic position (δ^15^N) compared to the prey items, which had 5.21 ‰ lower δ^15^N values than the mormyrid fish muscles in average (Figure 3; see Table S2 for raw values).

### 3.4 Calculations of trophic position within mormyrid genera

**FIGURE 4:**
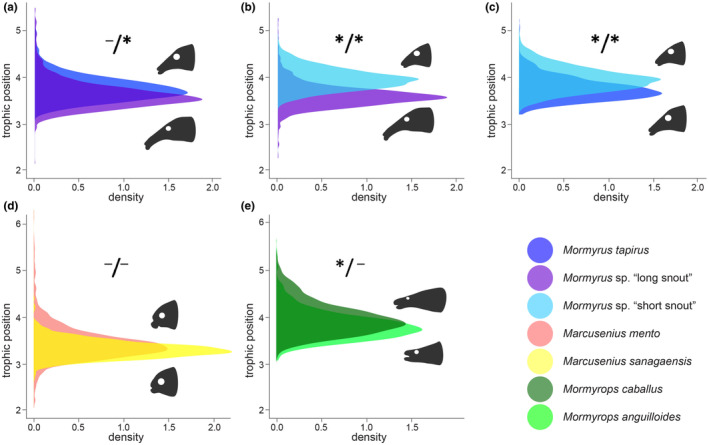
Density plots of trophic position (TP) comparing two species within one genus. (a–c) Three species of *Mormyrus* show larger differences among themselves, and in this case species with shorter protrusion tend to have a higher trophic position. (a) *Mormyrus tapirus* vs. *Mormyrus* sp. “long snout.” (b) *Mormyrus* sp. “long snout” vs. *Mormyrus* sp. “short snout,” (c) *Mormyrus tapirus* vs. *Mormyrus* sp. “short snout,” (d) *Marcusenius sanagaensis* vs. *Marcusenius mento*. (e) *Mormyrops anguilloides* vs. *Mormyrops caballus*. The baseline was generated from all benthic‐sourced samples from the prey samples. See Table 5 for the mean values of trophic position for all eleven species. The star/hyphen symbols refer to the results of independent pairwise *t*‐tests performed on the δ^13^C and δ^15^N values; the */* symbol means significant differences in both δ^13^C and δ^15^N, the ^−^/* symbol refers to significant difference only in the δ^15^N signal, */^−^ symbol refers to significant difference only in the δ^13^C signal and the −/− symbols refers to no significant difference (see Table 2 for exact values). Head shape projections shown for illustrative purposes. See Figure 6f for all species head shape diversity.

Mean trophic positions of all mormyrid species varied between 3 and 4 (Table 5). Pairwise comparisons within genera showed subtle differences in the trophic position of two species of *Mormyrops* (Figure 4e) and *Marcusenius* (Table 5, Figure 4d). On the other hand, we found larger differences among the three *Mormyrus* species, namely, *Mormyrus* sp. “short snout” showed a notable difference from *Mormyrus* sp. “long snout” and from *Mormyrus tapirus* (Table 2). Accordingly, an independent statistical analysis (*t*‐tests) on the δ^15^N and δ^13^C values revealed significant differences of the stable isotope values only in the *Mormyrus* species and between the δ^13^C values of the two *Mormyrops* species (see Table 2). It reflected nearly congruent trophic positions of the *Mormyrops* and the *Marcusenius* species, as well as the separation of *Mormyrus* sp. “short snout” from *Mormyrus tapirus* and from *Mormyrus* sp. “long snout.”

**TABLE 2:** Unequal variances *t*‐tests and Mann–Whitney‐*U*‐tests performed for delta nitrogen values as well as delta carbon values for species comparisons of the same genus (transition season samples excluded).

*t*‐test with different variances	*p*‐value (two‐tailed)
δ15N *Mormyrops caballus* vs. *Mormyrops anguilloides*	.650
δ15N *Marcusenius mento* vs. *Marcusenius sanagaensis*	.478
δ13C *Mormyrus tapirus* vs. *Mormyrus* sp.”short snout”	.001**
δ15N *Mormyrus tapirus* vs. *Mormyrus* sp.”short snout”	<.001***
δ13C *Mormyrus tapirus* vs. *Mormyrus* sp.”long snout”	.027
δ15N *Mormyrus tapirus* vs. *Mormyrus* sp.”long snout”	.002**
δ13C *Mormyrus* sp.”long snout” vs. *Mormyrus* sp.”short snout”	<.001***
δ15N *Mormyrus* sp. “long snout*”* vs. *Mormyrus* sp.”short snout”	<.001***
Mann–Whitney‐*U* test	
δ13C *Mormyrops caballus* vs. *Mormyrops anguilloides*	0.004**
δ13C *Marcusenius mento* vs. *Marcusenius sanagaensis*	0.092

**Siginificance level below 0.01; ***Below 0.001 (after Bonferroni correction in case of Mormyrus).

### 3.5 Seasonal differences in the isotopic signals

**FIGURE 5:**
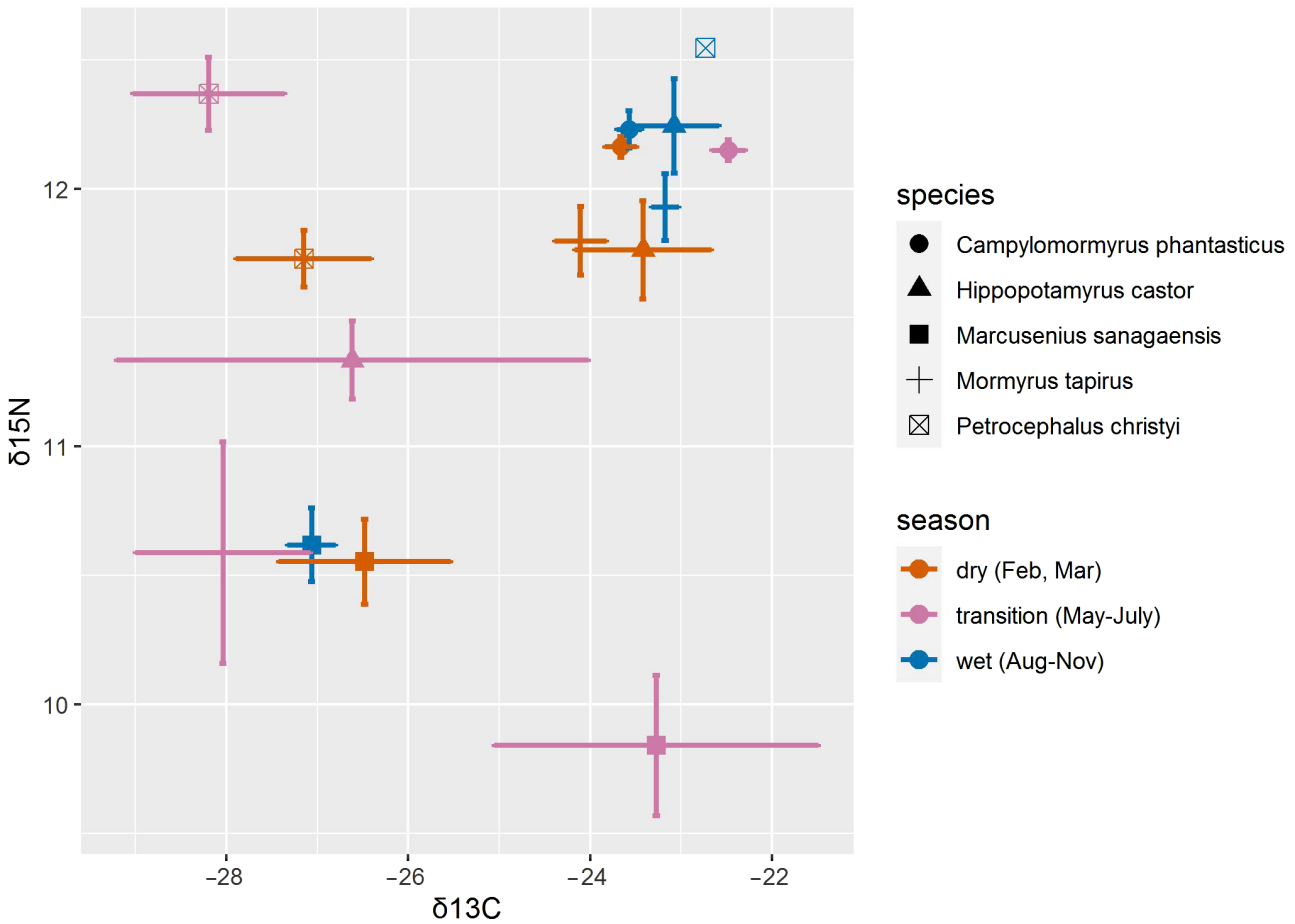
Isotopic bi‐plot (δ^13^C and δ^15^N; ‰) of five mormyrid species sampled across the wet (blue symbols), dry (red symbols) and transition (purple symbols) seasons. Horizontal and vertical error bars represent standard error around the mean. Note that only one sample was available for *Petrocephalus christyi* species collected during the wet season.

We investigated the influence of the season on the stable isotope values by comparing species sampled during the three seasons (Figure 5). Stable isotope values did not significantly differ between the dry and wet phases (Table 3), while differences were mostly observed between the transition phase and the two main seasons. Specifically, the wet‐vs‐transition and the dry‐vs‐transition phase differed for *Mormyrus tapirus* (in both δ^13^C and δ^15^N values), *Marcusenius sanagaensis* (only the δ^15^N values), and for *Campylomormyrus phantasticus* (only δ^13^C values in the dry vs. transition season). And the δ^15^N values differed between seasons for *Petrocephalus christyi*, but the analyses were not performed on all pairs as only one sample was available for the wet season. *Hippopotamyrus castor* showed no differences in the stable isotope signal among seasons (Table 3).

**TABLE 3:** Results of comparison tests (single‐factor ANOVAs and Kruskal–Wallis tests for all species with transition season samples.

Single‐factor ANOVA	*p*‐value	Df	Tukey‐HSD tests	Dry vs. wet	Dry vs. transition	Wet vs. transition
*Mormyrus tapirus* δ13C	<.001*	2		.197	*p* adj. < .001**	*p* adj. < .001**
*Mormyrus tapirus* δ15N	.001*	2		.844	.002**	.002**
*Marcusenius sanagaensis* δ15N	.011*	2		.961	.028**	.012**
*Petrocephalus christyi* δ15N dry vs. transition	.011*	1				
*Campylomormyrus phantasticus* δ15N	.654	2				
*Hippopotamyrus castor* δ13C	.232	2				
*Hippopotamyrus castor* δ15N	.169	2				
Kruskal–Wallis test			Dunn's test with Bonferroni adjusted *p*‐values	Dry vs. wet	Dry vs. transition	Wet vs. transition
*Marcusenius sanagaensis* δ13C	.115	2				
*Campylomormyrus phantasticus* δ13C	.027*	2		1.000	.024**	.082
*Petrocephalus christyi* δ13C dry vs. transition	.09	1				

* A *p*‐value below .05;

** Adjusted *p*‐value below .05.

### 3.6 Ecomorphology within the mormyrid species community

**FIGURE 6:**
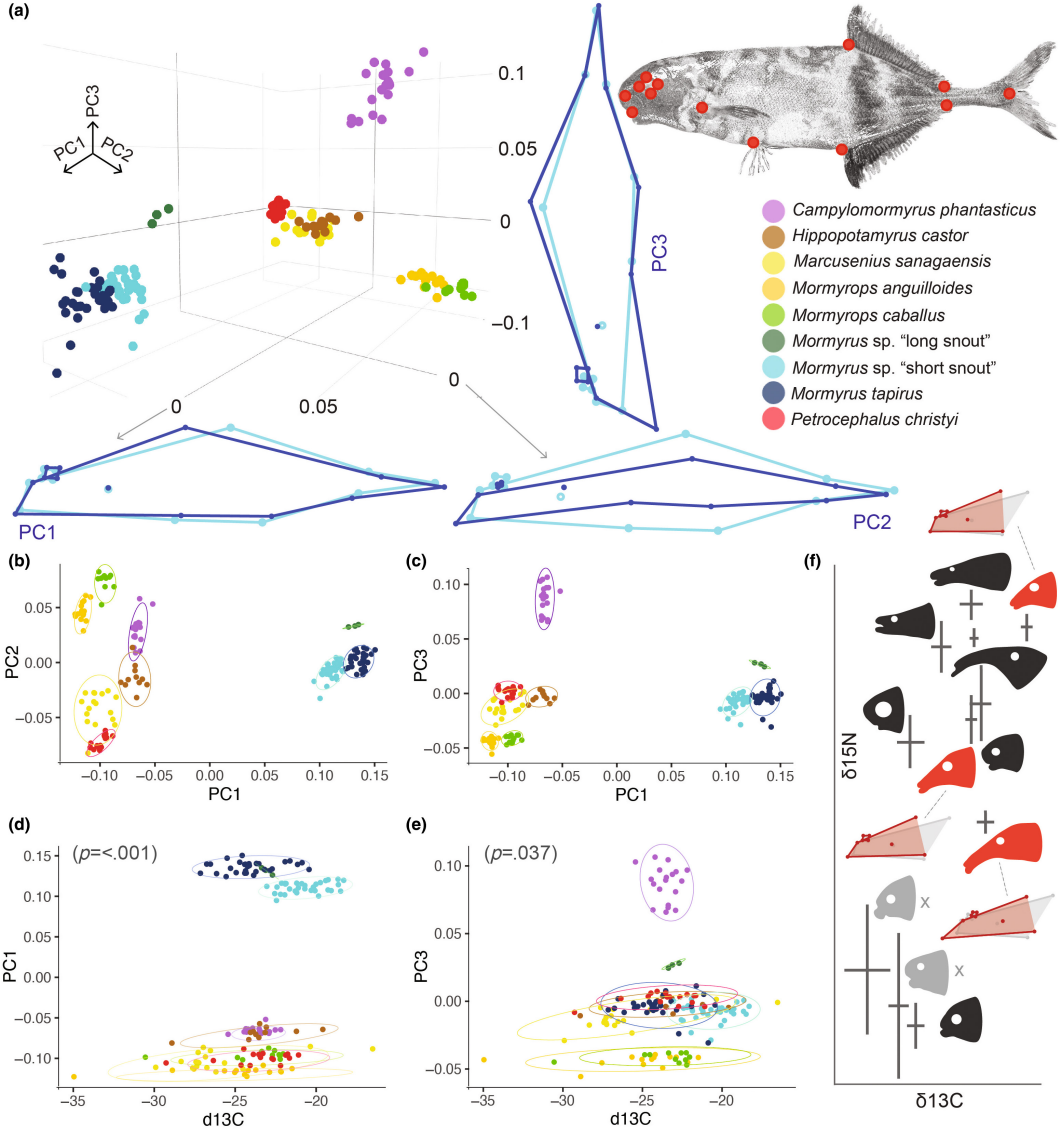
Ecomorphology of the mormyrid species community by geometric morphometrics. (a) The PCA of the overall body shape revealed clear separation of most of the species. Applied landmarks projected on the top right. The morphological changes along the three main axes (PC1, PC2, and PC3) are visualized as wireframes, light blue represents the average shape of all samples, and dark blue is the shape change along the respective axis. (b) PC1 against PC2 and (c) PC1 against PC3. Ellipses represent the confidence interval of 0.9. (d) The PLS analysis results for the PC1 and the δ^13^C values reveal a significant relationship (~overall shape and dorsal fin length; see the PC1 wireframes in Figure 6a). (e) PLS analysis for the PC3 (ca. snout shape/length) and δ^13^C shows an insignificant relationship after the Bonferroni correction. Note that only nine species could have been analyzed by the geometric morphometrics. (f) Simple head shape projection of all 11 species over the results of the stable isotope analysis (as in Figure 2a) to illustrate differences in snout protrusion. Two species missing in the geometric morphometric analysis marked by “X” symbol and shaded by light gray. Three species of the *Mormyrus* genus discussed in more detail are highlighted by bright red and their Procrustes average coordinates of the head shape (darker red) is projected over the all‐species average (gray). For the complete full‐body shape Procrustes coordinates of all nine species, please refer to Figure S6.

All nine species were clearly differentiated based on the overall morphometric analysis (Figure 6a–c; Figure S6). The first three PCA axes explained 93.9% of the total variance (PC1 = 75%, PC2 = 10.2% and PC3 = 8.7%). Shape variations along the PC1 axis were mostly driven by changes in body length and position of both the dorsal and anal fins. Shape variations associated with the PC2 axis were mostly explained by dorsoventral compression of body length and changes in eye size. The third PCA axis (PC3) reflected variations in snout length, a trait that is generally highly variable among mormyrid species (see exact shape variations along PCs axes in Figure 6a).

There was a significant association between the overall body shape (combined Procrustes coordinates) and the δ^13^C values (PLS regression; *p* < .001). To specify, we found a significant association between PC1 and δ^13^C values (PLS regression; RV = 0.118, *p* < .001, Table 6, Figure 6d), while PC2 and δ^13^C values were not significantly associated (Table 6). After Bonferroni correction, there was no significant relationship between PC3 and δ^13^C values (PLS regression; RV = 0.027, unadjusted *p* value = .037, Table 6). None of the PCs axes was associated with δ^15^N values (Table 6).

## 4 DISCUSSION AND CONCLUSIONS

### 4.1 Trophic niche partitioning in mormyrid fishes

Here, we provide the first comprehensive study of the mormyrid trophic ecology at the community level based on the stable isotope signals. Our results are in accord with previous evidence of mormyrids having fed mostly on the insect larvae (van der Waal, 1985; Winemiller & Adite, 1997). There are, however, noticeable differences among species, such as the two *Marcusenius* species and *P. batesii* having a lower trophic position than all other mormyrids (Figures 2a, 6f). This could be related to the known fact of *Marcusenius* being an omnivorous genus possibly feeding even on plant detritus, algae, or seeds at some locations (Adjibade et al., 2019). We further noticed differences in the niche sizes and overlaps as expected at the species community level. The smallest niche sizes were interestingly found in the species with the longest snouts: *Campylomormyrus phantasticus* and *Mormyrus* sp. “long snout” (Figure 2d) while the biggest niche size was found in *Petrocephalus christyi*. This was also reflected by the huge overlap and probability of finding other species in its own niche space (see Table 4). We have further specifically focused on the species of the same genus (i.e., evolutionary younger), and we found significant differences among three species of the *Mormyrus* genus. *Mormyrus tapirus* and two formally undescribed species, *M*. sp. “long snout,” and *M*. sp. “short snout,” differed significantly in δ^13^C and δ^15^N signals (see Table 2). Accordingly, *Mormyrus* sp. “short snout” also differed from the other two *Mormyrus* species in the trophic position analysis (Figure 4b,c). Our findings therefore suggest differential trophic specializations for the three different *Mormyrus* species. Trophic niche partitioning among closely related species from the same habitat has been previously observed in African fishes (e.g., Nagelkerke, 1997; Polačik et al., 2014), while resource partitioning has been a general question in ecology for a long time (Hardin, 1960; Schoener, 1974). The stable isotope analysis has been proven useful to visualize trophic niche partitioning within closely related fish species (Wang et al., 2022), and to our knowledge, our study is the first case of stable isotope analysis showing trophic niche partitioning in mormyrids.

**TABLE 4:** Probability calculations of pairwise comparisons to find species A in the niche region of species B; niche region defined as 95% probability region in multivariate space calculated using nicheROVER, Values for *Mormyrus* species pairwise comparisons in bold.

Species BSpecies A	*C. Phantasticus*	*H. Castor*	*Ma. Mento*	*Ma. Sanagaensis*	*Mo. Anguilloides*	*Mo. Caballus*	*Mu*. Sp. «long snout»	*Mu*. Sp. «short snout»	*Mu. Tapirus*	*Pa. Batesii*	*P. Christyi*
*C. phantasticus*	NA	99.94	20.60	25.09	99.97	91.96	0.05	43.87	99.83	2.31	100.00
*H. castor*	17.83	NA	22.34	51.86	80.85	37.56	0.89	26.77	74.32	14.51	88.88
*Ma. mento*	1.96	49.51	NA	84.40	54.71	7.11	0.05	1.16	19.33	41.48	82.33
*Ma. sanagaensis*	0.99	58.94	34.25	NA	34.79	2.94	0.28	1.03	41.46	44.70	55.86
*Mo. anguilloides*	16.71	78.90	23.24	33.64	NA	50.62	0.26	18.74	58.45	15.84	93.48
*Mo. caballus*	35.28	92.72	16.53	15.71	96.39	NA	0.03	43.30	75.18	7.80	98.32
*Mu*. sp. «long snout»	0.52	99.79	9.17	97.35	87.95	0.66	NA	**0.00**	**99.18**	1.76	98.94
*Mu*. sp. «short snout»	9.02	92.81	4.56	5.54	81.09	55.26	**0.01**	NA	**57.55**	0.36	95.84
*Mu. tapirus*	27.08	95.47	20.42	56.78	86.94	41.72	**1.28**	**25.27**	NA	11.29	91.83
*Pa. batesii*	0.23	63.46	50.20	92.39	47.06	5.55	0.01	0.11	27.21	NA	66.69
*P. christyi*	9.66	61.99	27.38	41.49	76.53	33.40	0.30	13.51	40.58	18.55	NA

*Note*: Note how even with *a* = 0.95 how little the probability is that any other species is found in the niche region of *Mormyrus* sp. “long snout”, whereas in contrast, the probability is drastically high for each species (except for *M. sanagaensis* and *P. batesii*) to be found in the isotopic niche of *Petrocephalus christyi*. Please note that the table reads by rows; for example, the probability of *C. phantasticus* (species A) to be found in the niche region of *Mormyrus* sp. “long snout” (species B) is 0.05, whereas the probability of *M*. sp. “long snout” (species A) to be found in the niche region of *C. phantasticus* (species B) is 0.52.

**TABLE 5:** Bayesian calculation of the trophic position by the tRophicPosition package in R. For pairwise comparison of TP density of multiple species in one genus, please refer to Figure 4.

Species	Mean TP	SD	SE
*C. phantasticus*	3.87	0.28	0.05
*H. castor*	3.90	0.27	0.04
*Marc. mento*	3.38	0.31	0.01
*Marc. sanagaensis*	3.32	0.24	0.01
*Mo. anguilloides*	3.83	0.27	0.01
*Mo. caballus*	3.91	0.26	0.02
*Mormyrus tapirus*	3.76	0.36	0.04
*Mormyrus* sp. *long snout*	3.69	0.56	0.07
*Mormyrus* sp. *short snout*	3.95	0.27	0.03
*Paramormyrops batesii*	3.35	0.36	0.01
*Petrocephalus christyi*	3.78	0.26	0.02

**TABLE 6:** Results of the PLS tests for relationship between the shape (geometric morphometrics) and isotopic signals.

Test	*p*‐value	Significance	RV
Procrust. coordinates vs. δ^13^C	<.001	***	0.144
PC1 vs. δ^13^C	<.001	***	0.118
PC2 vs. δ^13^C	.313		0.007
PC3 vs. δ^13^C	.037		0.027
Procrust. coordinates vs. δ^15^N	.503		0.024
PC1 vs. δ^15^N	.884		<0.001
PC2 vs. δ^15^N	.931		<0.001
PC3 vs. δ^15^N	.254		0.009

*Note*: Unadjusted *p*‐value shown; ***Stands for significance below 0.001 after Bonferroni correction; as well as RV coefficient shown.

### 4.2 Ecomorphology: Body shape in relation to the trophic ecology

Differences in trophic preferences can be reflected on the body morphology, such as jaw/mouth shape, which is often adapted to its specific purpose. For example, in croakers (Sciaenidae), the species with the most unique isotopic signatures also had a unique feeding strategy associated with different jaw shape (Zhang et al., 2020). Previous (eco)morphological studies in mormyrids have focused either on a smaller scale of one genus (*Campylomormyrus;* Lamanna et al., 2016 and Feulner et al., 2007 and 2008, and *Paramormyrops*; Arnegard et al., 2010) or on a much broader level to test convergence between Mormyridae and Apteronotidae (Ford et al., 2022). Applying geometric morphometrics, we found a clear separation of the species by morphology in general (Figure 6a). These findings were similar to those previously found in *Campylomormyrus* (Lamanna et al., 2016). Unfortunately, two species with short snout (*M. mento* and *P. batesii*) were not used for geometric morphometric analyses due to the lack of photographs. These species had (together with *M. sanagaensis*) the lowest δ^13^C and δ^15^N values and hence the variability of the dataset was reduced a lot by their exclusion. Without these two species, a relationship between the snout shape/length itself (PC3) and the δ^13^C values is insignificant (Figure 6e), and we would need more complete sampling to properly test the relationship between ecology and snout morphology in the future. Notably, the three *Mormyrus* species have different isotopic signals and at the same time they also express anatomical differences in their trunk shape and jaw and lip morphology, which unfortunately could not be reflected by our geometric morphometric analysis (since our landmarks could not cover such details due to the photographs limitations). *Mormyrus* sp. “short snout” had a more truncated upper lip protrusion while *M. tapirus* had a longer lower lip, and *M*. sp. “long snout” had a much longer snout than the two other *Mormyrus* species (Figures 2c, 6f). The overall mouth shape and orientation probably reflect subtle differences in microhabitat and prey preferences in these species. Our data showed the differential trophic specialization, and this could then possibly be associated with the snout shape differences on the shorter evolutionary scale, that is, in the three closely related species. This seems to be in accord with the hypothesis of Feulner et al. (2007 and 2008), who suggested variations in trophic ecology based on snout morphological differences in *Campylomormyrus* from the lower Congo River.

### 4.3 Phylogenetic relationships among Sanaga mormyrids

We reconstructed evolutionary relationships among the eleven mormyrids species from Sanaga, and our phylogenetic hypothesis is in accord with previous phylogenies (Carlson et al., 2011; Lavoué et al., 2000; Sullivan et al., 2000, 2002), namely, by showing the genus *Marcusenius* as not monophyletic. Albeit our phylogeny includes just 11 species found in Sanaga, we can briefly conclude that elongated snout (*Mormyrus*, *Campylomormyrus*) or rounded head (*Petrocephalus*, *Hippopotamyrus*) have evolved several times in unrelated mormyrid species. Similarly, the results of trophic and isotopic niches did not seem to carry any simple or strong phylogenetic signal, although we did not specifically test for this. Future comparative studies, including more species, could investigate this pattern more thoroughly. Most likely, the phylogenetic signal gets overwritten by repeated competition within the ecological opportunity window resulting in niche specializations. Certainly, microhabitat and behavior differences in feeding play a role as hypothesized for three *Campylomormyrus* species with differing habitat preferences (Amen et al., 2020), and the same likely applies also to other genera, such as *Mormyrus* and *Mormyrops* in this study. Finally, we confirmed the identity of the two undescribed species, *Mormyrus* sp. “short snout” and *Mormyrus* sp. “long snout,” and these species belong to the genus *Mormyrus*. This evidence allowed us to make the aforementioned conclusions about the closely related species.

### 4.4 Prey samples, baseline, and effect of turnover rates on the analysis

The environmental benthic samples were set as a baseline for the analysis; however, it has to be acknowledged that its power is limited as the prey samples were not derived from all localities. We also used stomach content samples to calculate trophic positions, which resulted in very similar results (see Supplementary Material S3). The prevalence of different insects found in the stomachs and used for the baseline reconstruction corresponds to the previous studies on the trophic preferences of mormyrids (e.g., Adjibade et al., 2019; van der Waal, 1985; Winemiller & Adite, 1997). Working on an understudied group of tropical fishes, such as mormyrids, may be challenging as other factors contribute to the final patterns for example in our case, seasonal changes (possible migration), differing turnover rates, or unknown discrimination factors for Bayesian analysis. Turnover rates are not known for mormyrids, and based on other species, generally they are two to 3 months in muscle tissue or bone collagen (while just few days in plasma or blood; e.g., Madigan et al., 2021; Polischuk et al., 2001). There are hints that the turnover rates can also differ among species, fish size, and life stage (Bosley et al., 2002; Gaston & Suthers, 2004; Madigan et al., 2012; Madigan et al., 2021).

Importantly, discrimination factor values can differ depending on the diet and consumers feeding on protein‐enriched diet have been reported to have a higher discrimination factor than those feeding exclusively on invertebrates (McCutchan Jr et al., 2003). Here, the minimum trophic discrepancy for δ^15^N was 2.5‰ between mormyrids and their prey (Figure 3), suggesting that most mormyrid species have a protein‐enriched diet. In the future, our results could be complemented by a long‐term stomach content analyses like in Davis et al. (2012), Layman et al. (2012), Polačik et al. (2014), or Dillon et al. (2021). Furthermore, future studies could use a more thorough prey samples collection from all localities to achieve a more accurate baseline of prey samples (Post, 2002), which would allow application of more sophisticated mixed models for diet reconstruction.

### 4.5 Seasonality in the isotopic signal

Dry and wet seasons in tropical regions can be reflected in fish behavior, migration, reproduction, or food/prey availability. The impact of the season on the stable isotope values has previously been reported from other fish groups (McMeans et al., 2019; Nashima et al., 2020; Taylor, 2016; Wantzen et al., 2002), and seasonal variability seems to be especially relevant for partly flooded aquatic ecosystems (Taylor et al., 2017; Merron & Mann, 1995; de Almeida et al., 1997; Wantzen et al., 2002; eggleton & schramm, 2004). The isotopic signal can be influenced by various factors, not only by the actual changes in the prey availability. Recent studies revealed that some fish become more generalist feeders during the wet season (Costa‐Pereira et al., 2017; pool et al., 2017) or have a higher trophic position during the dry season (Wantzen et al., 2002), whereas other studies reported diverse foraging strategies with no seasonal influence (McMeans et al., 2019; Novakowski et al., 2008). We found differences in isotopic signal between the transition phase (May–July) compared to both the wet season (August–November) and the dry season (February–March), whereas no difference was found between dry and wet season. The different isotopic signals were present in four out of five tested species (*Mormyrus tapirus*, *Marcusenius sanagaensis, Petrocephalus christyi*, and *Campylomormyrus phantasticus*, while *Hippopotamyrus castor* did not show any differences throughout seasons). Therefore, the transition season seems to influence the diet of some mormyrid species, although the reason for this is unknown. The habitats of the Sanaga River and its tributaries are characterized by seasonal flooding during the wet season and are exposed to a rising water level already in the transition phase. Multiple factors could contribute to the observed seasonality differences. First, the individuals sampled during the three seasons could originate from different populations as seasonal migration of mormyrid species are commonly observed during the wet season (Blake, 1977; Okedi, 1965; Olopade, 2013; Van der Waal, 1996). However, virtually nothing is known about migration of mormyrids in the Sanaga River. One of the species, *Marcusenius sanagaensis*, is known to prefer the tributaries over the main stream (Njom et al., personal observations based on several years of fieldwork). Alternatively, turnover rates of a few months (for white muscle tissue in other studied fish species; Taylor et al., 2017) could mean that the transition season values actually reflect a change of diet during the dry season. Interestingly, this could also be an anthropogenic effect due to the phytosanitary treatment of the plantations along the tributaries carried out toward the end of the major dry season (end of January until the end of February; Njom et al., 2022). Such unusually large terrestrial input to the water may possibly impact the signal in some (but not all) species. Lastly, we can also explain the pattern partially by different sampling locations in the transition period for some species, but for some, the results stay the same irrespective of the locality (Table S1). For example, the δ^15^N samples of *Marcusenius sanagaensis* from the Mekono River (a tributary with a pollution impact largely increased during the dry season) were different from the main Sanaga River, suggesting that the isotopic signals of *Marcusenius sanagaensis* were more influenced by locality than changes in seasonality (Table S3, Figure S4a). In *Mormyrus tapirus*, season and locality seem to both play a role. The difference in wet vs. transition δ^13^C values (but not in δ^15^N) remains after exclusion of the samples from Mpem River (see Figure S4c) yet only for unadjusted *p*‐values (Table S3). Interestingly, the transition samples from the same locality (Mpem River) were not the ones causing the significant difference in *Marcusenius sanagaensis*, which points again to differences among species. Some differences of the transition samples like the δ^15^N values of *Petrocephalus christyi* remain in the results irrespective of locality or could not be examined as they were from one locality (the δ^13^C values of *Campylomormyrus phantasticus*). Unfortunately, our sampling was not robust enough to test properly the effects of all aforementioned factors possibly involved in the seasonally different signal of the stable isotopes in mormyrids. To acknowledge also the possible overall effect of locality due to having sampled two further located streams within the same Sanaga River system (Figure 1, Park and Sanaga stream), an additional analysis was conducted where all Park stream locality samples are excluded from the dataset (Figure S5). Interestingly, the δ^15^N values did not change, or only subtly (minus 0.1 ‰), whereas the δ^13^C values were slightly more affected by the exclusion of the tributary localities (minus 0.1–0.4‰). Noteworthy, the strongest impact was found in the species *Petrocephalus christyi* (minus 1.6‰ in δ^13^C) so they get even more depleted in 13C (Figure S4b). These findings again highlight the need for further and more thorough studies on the individual fish species and their migration patterns.

To conclude, here we present the first comprehensive study applying stable isotope analysis on mormyrid fishes at a species community level. We found differences in the isotopic signal among eleven species corresponding to feeding specialization, and we also reported a correlation between body shape and trophic ecology (δ^13^C values) of different mormyrid species. We further specifically focused on the isotopic differences between three *Mormyrus* species with clear trophic niche differentiation after their recent evolution. Lastly, we report on seasonal differences in the isotopic signal in four species, which remains to be further investigated regarding its consistency and cause. Our study attempts to shed light on the biology of the evolutionarily successful and diverse—and yet highly understudied—group of African tropical freshwater fishes.

## AUTHOR CONTRIBUTIONS

**Gina Maria Sommer:** Data curation (lead); formal analysis (lead); investigation (equal); methodology (equal); visualization (equal); writing – original draft (lead); writing – review and editing (equal). **Samuel Didier Njom:** Data curation (equal); formal analysis (supporting); investigation (equal); resources (equal); writing – review and editing (equal). **Adrian Indermaur:** Conceptualization (equal); investigation (equal); supervision (supporting); writing – review and editing (equal). **Arnold Roger Bitja Nyom:** Conceptualization (equal); project administration (equal); resources (equal); supervision (equal); writing – review and editing (equal). **Kateřina Jandová:** Data curation (equal); resources (equal); writing – review and editing (supporting). **Jaroslav Kukla:** Data curation (equal); resources (equal); writing – review and editing (supporting). **Miloslav Petrtýl:** Formal analysis (equal); methodology (supporting); visualization (supporting); writing – review and editing (supporting). **Petra Horká:** Conceptualization (equal); data curation (equal); formal analysis (supporting); funding acquisition (supporting); methodology (equal); resources (equal); supervision (equal); writing – review and editing (equal). **Zuzana Musilova:** Conceptualization (lead); funding acquisition (lead); project administration (lead); resources (equal); supervision (lead); visualization (equal); writing – original draft (lead); writing – review and editing (equal).

## FUNDING INFORMATION

The project has been funded by the Swiss National Science Foundation (PROMYS–166,550) and the PRIMUS Research Programme (Charles University). ZM was further supported by the Czech Science Foundation (21‐31712S). GMS was supported by GAUK (251299). PH was further supported by a grant of the Czech Health Research Council (AZV CR, number NU21‐04‐00405).

## CONFLICT OF INTEREST STATEMENT

The authors declare no conflict of interests.

## Supporting information


Figure S1.
Figure S2.
Figure S3.
Figure S4.
Figure S5.
Figure S6.

Table S1.
Table S2.
Table S3.

## Data Availability

COI sequences used for the species phylogeny are available on GenBank for *Marcusenius mento* with accession numbers: HM882757 and HM882727, and the FASTA sequences for the other species are available in the Supplementary Material. COI sequences sequenced within this study are also available at GenBank (Accession numbers: PP776773 ‐ PP776792). Raw data of stable isotope analysis of the mass spectrometer for muscle samples are imbedded in sample overview table in Table S1 in Supporting information and for trophic samples in Table S2.
