# Supplementary material for: Trophic ecology of the African riverine elephant fishes (Mormyridae)
Source: Ecol Evol. 2024 Aug 27;14(8):e70173. doi: 10.1002/ece3.70173 (PMC11349487; doi:10.1002/ece3.70173)
Supplement: Supplementary file 1 — Figure S1. Figure S2. Figure S3. Figure S4. Figure S5. Figure S6. [file ECE3-14-e70173-s002.docx]

Supplementary Material for: **Trophic ecology of the African riverine elephant fishes (Mormyridae)**

**Figure S1: Stable isotope analysis of the prey samples from the benthic samples**

**
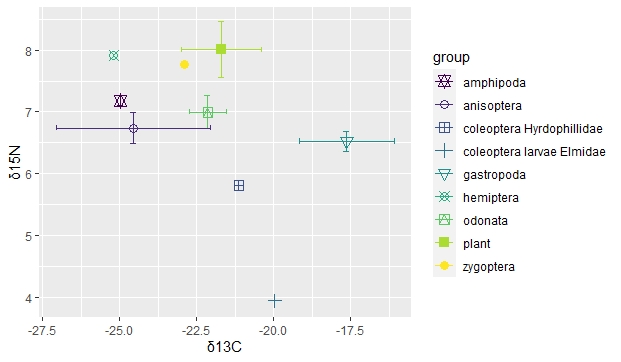
**

**Figure S2: Stable isotope analysis of the prey samples from the stomach contents**

**
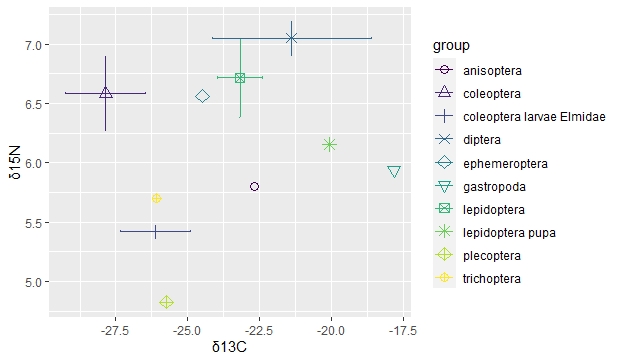
**

**Figure S3: Trophic positions comparisons based on stomach content baseline**

**
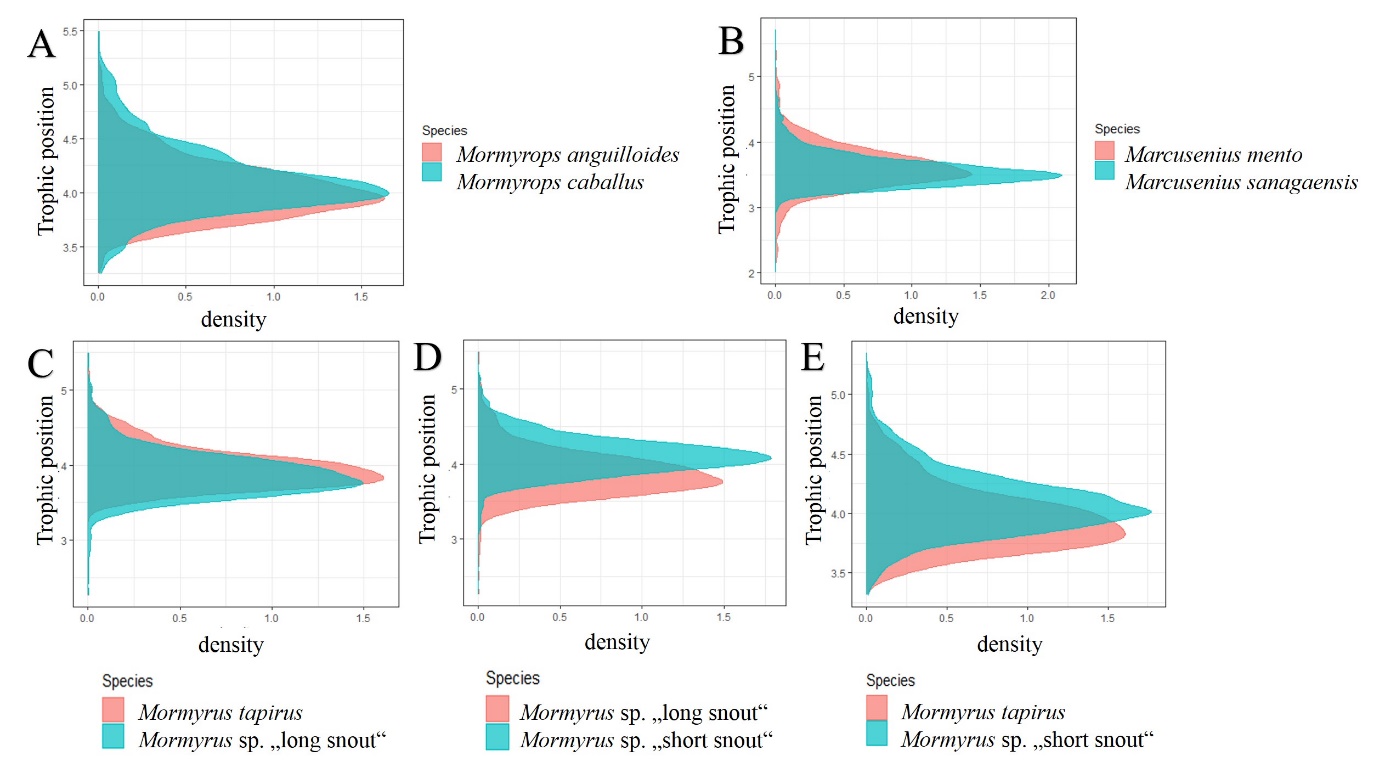
**

**Figure S4: *Marcusenius sanagaensis* (A) and *Petrocephalus christyii* (B) samples divided by locations and (C) *Mormyrus tapirus* divided by season (arrows indicate transition samples not from Mpem)**


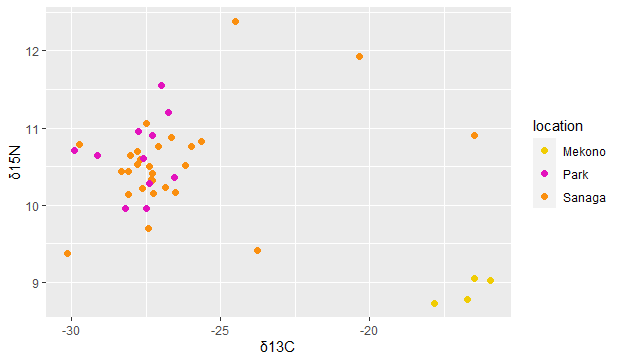

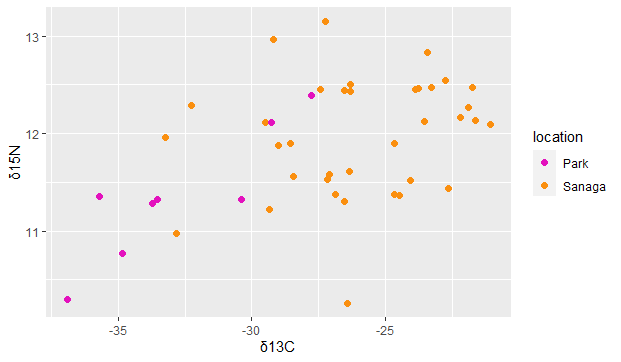


B

A

C


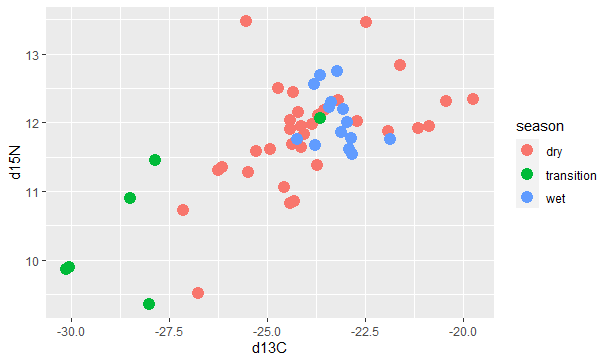


**Figure S5: Results of the stable isotope analysis of the muscle samples without the Park stream samples- mean and standard errors shown.**

**
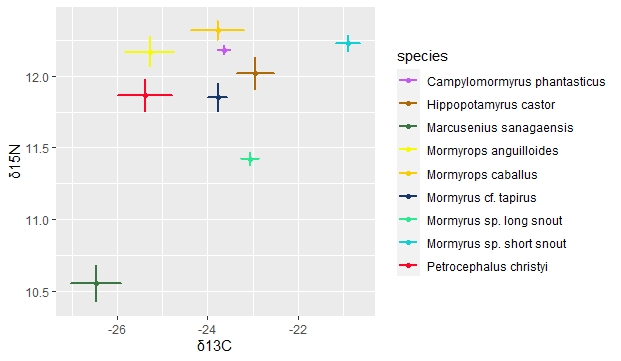
**

Please note that *Marcusenius mento* and *Paramormyrops batesii* are missing due to only having Park stream derived samples

**
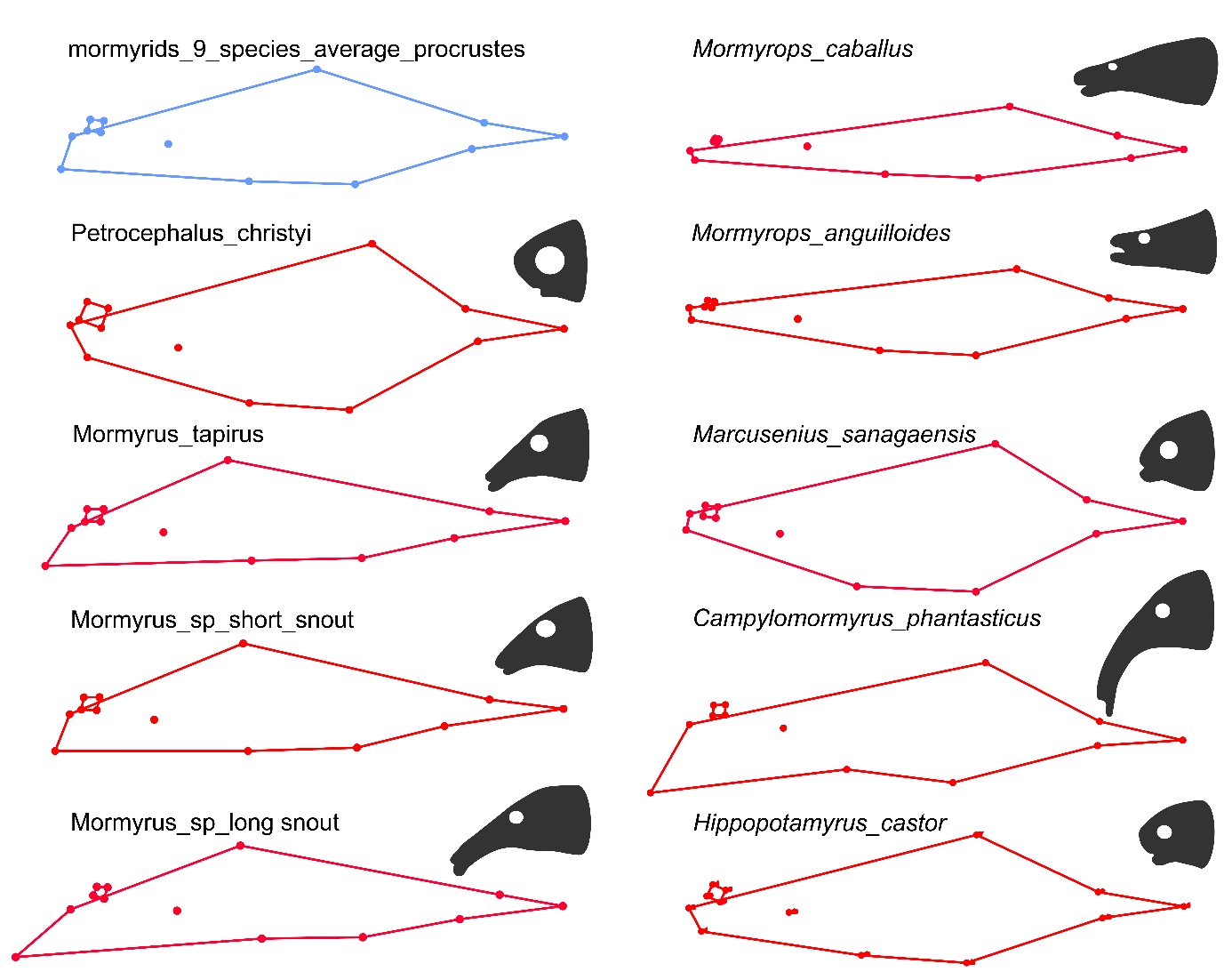
Figure S6: Procrustes average coordinates of body shape for all nine tested species**

**Table S1: Sample list overview of muscle samples isotope values and details about sampling**

**Table S2: Sample list overview of prey samples**

**Table S3: Results of comparison tests (single-factor ANOVAs and Kruskal-Wallis tests)** **for species with transition season samples testing the factor of locality**

Provided together in a separate pdf file

**COI FASTA sequences:**

>52A1_Mormyrus_sp_short_snout

GCGCTAAGCCTCCTAATTCGAGCCGAACTAAACCAACCTGGGGCACTACTTGGTGACGACCAGATTTATAATGTCATCGTCACAGCACACGCCTTCGTAATAATTTTCTTCATGGTAATACCAATTATAATTGGCGGATTTGGCAACTGACTAATTCCTCTCATGCTAGGCGCCCCCGACATAGCATTCCCCCGAATAAATAACATAAGCTTCTGACTCCTACCCCCATCATTCCTGCTCTTACTCGCCTCCTCTGGCGTAGAGGCCGGGGTGGGAACCGGGTGAACTGTCTACCCACCGCTGGCCGGTAACCTGGCCCACGCAGGGGCCTCCGTAGACCTGGCTATTTTCTCCCTCCACCTAGCCGGAGTATCATCTATCCTAGGCTCAATCAACTTCATCACCACAATTATCAATATGAAACCGCCAGCAACCTCCCAATACCAGACCCCCCTCTTCATCTGGGCTACTTTAGTAACCACAGTCCTGCTGCTGCTATCGCTGCCAGTACTTGCCGCAGGAATTACAATACTATTAACGGACCGAAACCTGAACACAACATTCTTTGACCCAGCAGGAGGAGGAGACCCAATTCTCTACCAACACTTATTC

>52B5_Marcusenius_sanagaensis Marcusenius sanagaensis

GCACTAAGCCTACTCATCCGAGCGGAACTAAACCAACCTGGGGCCCTGCTTGGAGATGACCAGATTTATAATGTTATCGTCACAGCACACGCCTTCGTAATAATTTTCTTCATGGTAATGCCCATTATGATCGGTGGCTTCGGCAACTGATTAATTCCCCTCATACTAGGCGCCCCTGACATGGCATTCCCTCGAATAAATAACATAAGTTTCTGGCTCCTGCCCCCCTCATTCCTTCTTCTACTAGCCTCTTCTGGCGTAGAGGCTGGGGTTGGTACGGGATGAACCGTCTACCCCCCGCTAGCCGGCAACCTTGCCCATGCTGGAGCCTCCGTAGACCTAGCCATTTTCTCTCTTCACCTGGCCGGAGTCTCCTCCATCCTTGGTTCAATCAACTTTATTACCACAATTATCAACATAAAACCCCCAGCAATTTCTCAATATCAAACCCCATTATTCATTTGAGCCCTGCTAGTTACTACTGTGCTTCTGCTGCTGTCATTACCAGTTTTAGCTGCAGGAATTACGATGCTACTAACAGATCGAAACCTAAACACAACATTTTTTGACCCGGCAGGCGGGGGGGATCCAATCCTCTACCAACACTTATTC

>52G5_Petrocephalus_christyi Petrocephalus christyi

GCACTGAGCCTCCTGATCCGAGCAGAACTAAACCAACCTGGGGCCTTGCTTGGCGACGACCAGATTTATAATGTCATCGTTACTGCACATGCCTTCGTAATAATTTTCTTCATGGTAATACCAATTATAATCGGAGGGTTTGGCAACTGACTAATTCCACTCATGCTAGGCGCCCCAGACATGGCGTTCCCCCGAATAAATAACATAAGCTTCTGACTACTACCCCCATCCTTCCTCCTTCTCCTTGCTTCTTCAGGAGTAGAGGCCGGGGTTGGAACCGGCTGAACGGTCTACCCACCATTGGCCAGTAATTTAGCCCATGCCGGGGCCTCTGTTGACCTAGCCATCTTTTCTCTTCACCTGGCAGGAGTGTCCTCTATTTTAGGCTCAATCAACTTCATCACCACAATTATTAATATAAAACCACCAGCAATTTCCCAATACCAAACCCCACTATTCATCTGGTCCCTGCTAGTAACCACAGTACTACTCCTACTTTCCCTACCAGTCCTGGCTGCAGGCATTACTATATTATTAACAGACCGAAACCTAAACACAACATTCTTCGACCCAGCCGGAGGGGGAGACCCAATTCTATATCAACACCTCTTC

>60D8_Mormyrus_cf_tapirus Mormyrus tapirus

GCRCTGAGCCTCCTAATTCGAGCCGAACTAAACCAACCTGGGGCACTGCTTGGTGACGACCAGATTTATAATGTCATCGTCACAGCACACGCCTTCGTAATAATTTTCTTCATGGTAATACCAATTATAATTGGCGGATTCGGCAACTGACTAATTCCTCTCATGCTGGGCGCCCCCGACATGGCATTCCCCCGAATAAACAACATAAGCTTCTGACTCCTGCCCCCATCATTCCTGCTCTTGCTCGCCTCCTCTGGCGTGGAGGCCGGGGTCGGAACCGGATGAACTGTCTACCCACCCCTGGCCGGTAACCTGGCCCACGCAGGGGCCTCCGTAGACCTGGCTATTTTCTCCCTCCACCTAGCCGGAGTATCATCTATCCTAGGCTCAATCAACTTCATCACCACAATTATCAATATGAAACCACCAGCGATCTCCCAATACCAGACCCCTCTCTTCATCTGGGCTACTTTAGTAACCACAGTCCTGCTACTGCTATCGCTGCCAGTCCTCGCCGCAGGAATTACAATGCTGCTAACGGACCGAAACCTAAACACAACRTTCTTTGACCCAGCAGGCGGAGGAGACCCAATCCTCTACCAACACCTATTC

>60E4_Hippopotamyrus_castor Hippopotamyrus castor

GCACTAAGCCTTTTAATCCGAGCAGAACTAAATCAACCTGGAGCCCTACTTGGGGACGACCAGATTTATAATGTTATTGTTACAGCACACGCCTTCGTAATAATTTTCTTCATGGTAATACCAATTATGATCGGTGGCTTCGGCAACTGGCTAATCCCACTTATACTTGGCGCCCCTGACATAGCCTTCCCCCGAATAAATAATATAAGCTTCTGACTTCTACCCCCATCATTCCTTCTTCTGCTAGCCTCCTCTGGTGTAGAAGCTGGAGCTGGAACTGGGTGAACCGTCTACCCCCCATTAGCCGGCAACCTCGCCCATGCTGGAGCTTCCGTAGACCTGGCTATTTTCTCCCTGCACCTAGCTGGAATCTCTTCTATTCTTGGCTCAATCAACTTTATCACAACAATTATTAATATAAAACCCCCAGCAATCTCTCAATACCAAACCCCACTTTTTATTTGAGCTTTATTAGTAACCACTGTACTCCTACTACTATCCCTCCCAGTCCTAGCTGCAGGTATCACAATACTATTAACAGACCGAAACCTAAACACAACCTTCTTTGATCCCGCAGGCGGGGGAGACCCAATCCTTTATCAACACTTATTC

>60E5_Hippopotamyrus_castor Hippopotamyrus castor

GCACTAAGCCTTTTAATCCGAGCAGAACTAAATCAACCTGGAGCCCTACTTGGGGACGACCAGATTTATAATGTTATTGTTACAGCACACGCCTTCGTAATAATTTTCTTCATGGTAATACCAATTATGATCGGTGGCTTCGGCAACTGGCTAATCCCACTTATACTTGGCGCCCCTGACATAGCCTTCCCCCGAATAAATAATATAAGCTTCTGACTTCTACCCCCATCATTCCTTCTTCTGCTAGCCTCCTCTGGTGTAGAAGCTGGAGCTGGAACTGGGTGAACCGTCTACCCCCCATTAGCCGGCAACCTCGCCCATGCTGGAGCTTCCGTAGACCTGGCTATTTTCTCCCTGCACCTAGCTGGAATCTCTTCTATTCTTGGCTCAATCAACTTTATCACAACAATTATTAATATAAAACCCCCAGCAATCTCTCAATACCAAACCCCACTTTTTATTTGAGCTTTATTAGTAACCACTGTACTCCTACTACTATCCCTCCCAGTCCTAGCTGCAGGTATCACAATACTATTAACAGACCGAAACCTAAACACAACCTTCTTTGATCCCGCAGGCGGGGGAGACCCAATCCTTTATCAACACTTATTC

>60E6_Mormyrus_sp_long_snout

GCACTGAGCCTCCTAATTCGAGCCGAACTAAACCAACCTGGGGCACTGCTTGGTGACGACCAGATTTATAATGTCATCGTCACAGCACACGCCTTCGTAATAATTTTCTTCATGGTAATACCAATTATAATTGGCGGATTTGGCAACTGACTAATTCCTCTCATGCTAGGCGCCCCCGACATGGCATTCCCACGAATAAATAACATAAGCTTCTGACTCCTACCCCCATCATTTCTGCTATTGCTCGCCTCCTCTGGCGTGGAGGCCGGGGTCGGAACCGGGTGAACTGTCTACCCACCCCTAGCCGGCAACCTGGCCCACGCAGGGGCCTCCGTAGACCTGGCCATTTTCTCCCTCCACCTAGCCGGAGTATCATCTATCCTAGGCTCAATCAACTTCATCACCACAATTATCAACATGAAACCACCAGCGATCTCCCAATACCAGACCCCTCTCTTCATCTGGGCCACTTTAGTAACCACAGTCCTGCTACTGCTATCGCTGCCAGTCCTCGCCGCAGGAATTACAATGCTGTTAACAGACCGAAACCTAAACACAACATTCTTTGACCCGGCAGGAGGAGGAGACCCAATTCTCTACCAACACCTATTC

>60E7_Mormyrus_sp_long_snout

GCACTGAGCCTCCTAATTCGAGCCGAACTAAACCAACCTGGGGCACTGCTTGGTGACGACCAGATTTATAATGTCATCGTCACAGCACACGCCTTCGTAATAATTTTCTTCATGGTAATACCAATTATAATTGGCGGATTTGGCAACTGACTAATTCCTCTCATGCTAGGCGCCCCCGACATGGCATTCCCACGAATAAATAACATAAGCTTCTGACTCCTACCCCCATCATTTCTGCTATTGCTCGCCTCCTCTGGCGTGGAGGCCGGGGTCGGAACCGGGTGAACTGTCTACCCACCCCTAGCCGGCAACCTGGCCCACGCAGGGGCCTCCGTAGACCTGGCCATTTTCTCCCTCCACCTAGCCGGAGTATCATCTATCCTAGGCTCAATCAACTTCATCACCACAATTATCAACATGAAACCACCAGCGATCTCCCAATACCAGACCCCTCTCTTCATCTGGGCCACTTTAGTAACCACAGTCCTGCTACTGCTATCGCTGCCAGTCCTCGCCGCAGGAATTACAATGCTGTTAACAGACCGAAACCTAAACACAACATTCTTTGACCCGGCAGGAGGAGGAGACCCAATTCTCTACCAACACCTATTC

>60F1_Mormyrops_anguilloides Mormyrops anguilloides

GCACTGAGCCTCCTTATCCGAGCGGAACTAAACCAACCAGGGGCCCTGCTTGGCGACGACCAGATTTATAATGTTATCGTTACAGCACACGCCTTCGTAATAATTTTCTTCATAGTAATGCCAATCATGATCGGCGGTTTTGGCAACTGATTAATCCCCCTCATGCTCGGCGCCCCAGATATAGCATTTCCCCGAATGAACAACATAAGCTTTTGACTCCTACCACCATCCTTCCTCCTTCTACTTGCCTCTTCTGGAGTCGAAGCTGGAGTTGGGACAGGATGAACTGTCTACCCGCCCCTGGCGGGCAATTTAGCCCATGCCGGAGCCTCTGTAGACCTGGCCATTTTCTCCCTCCACTTAGCCGGTGTGTCCTCTATCCTTGGCTCTATCAACTTCATTACCACAATCATCAACATAAAACCCCCAGCAATCTCCCAATACCAAACCCCCCTTTTTATTTGAGCCCTATTAGTGACCACCGTCTTACTACTACTATCCCTGCCAGTCCTAGCTGCAGGAATCACAATACTACTAACAGATCGGAACCTGAATACAACATTCTTCGACCCGGCAGGCGGAGGAGACCCAATCCTCTACCAACACCTATTC

>60F3_Mormyrus_sp_short_snout

GCGCTAAGCCTCCTAATTCGAGCCGAACTAAACCAACCTGGGGCACTACTTGGTGACGACCAGATTTATAATGTCATCGTCACAGCACACGCCTTCGTAATAATTTTCTTCATGGTAATACCAATTATAATTGGCGGATTTGGCAACTGACTAATTCCTCTCATGCTAGGCGCCCCCGACATAGCATTCCCCCGAATAAATAACATAAGCTTCTGACTCCTACCCCCATCATTCCTGCTCTTACTCGCCTCCTCTGGCGTAGAGGCCGGGGTGGGAACCGGGTGAACTGTCTACCCACCGCTGGCCGGTAACCTGGCCCACGCAGGGGCCTCCGTAGACCTGGCTATTTTCTCCCTCCACCTAGCCGGAGTATCATCTATCCTAGGCTCAATCAACTTCATCACCACAATTATCAATATGAAACCGCCAGCAACCTCCCAATACCAGACCCCCCTCTTCATCTGGGCTACTTTAGTAACCACAGTCCTGCTGCTGCTATCGCTGCCAGTACTTGCCGCAGGAATTACAATACTATTAACGGACCGAAACCTGAACACAACATTCTTTGACCCAGCAGGAGGAGGAGACCCAATTCTCTACCAACACTTATTC

>60H1_Mormyrops_caballus Mormyrus caballus

GCACTAAGCCTCCTTATCCGAGCGGAACTAAACCAACCGGGGGCCCTGCTTGGCGACGACCAGATTTATAATGTTATCGTCACAGCACACGCCTTCGTAATAATTTTCTTCATAGTAATGCCAATCATGATCGGCGGTTTTGGCAACTGATTAATCCCCCTCATGCTCGGCGCCCCAGATATAGCATTTCCCCGAATGAACAACATAAGCTTTTGACTCCTACCACCATCCTTCCTCCTTCTACTTGCCTCCTCTGGAGTCGAAGCTGGAGTTGGGACAGGATGAACTGTCTACCCGCCCCTGGCGGGCAATTTAGCCCATGCCGGAGCCTCTGTAGACCTGGCTATTTTCTCCCTCCACTTAGCCGGTGTATCCTCCATCCTTGGCTCTATCAACTTCATCACCACAATCATCAACATAAAACCCCCAGCAATCTCCCAATACCAAACCCCCCTTTTTATTTGAGCCCTATTAGTGACCACCGTCTTACTACTACTATCCCTGCCAGTCCTAGCTGCAGGAATCACAATACTACTAACAGATCGGAACCTGAATACAACATTCTTCGACCCAGCAGGCGGAGGAGACCCAATCCTCTACCAACACCTATTC

>75A4_Marcusenius_sp Marcusenius sanagaensis

GCACTAAGCCTACTCATCCGAGCGGAACTAAACCAACCTGGGGCCCTGCTTGGAGATGACCAGATTTATAATGTTATCGTCACAGCACACGCCTTCGTAATAATTTTCTTCATGGTAATGCCCATTATGATCGGTGGCTTCGGCAACTGATTAATTCCCCTCATACTAGGCGCCCCTGACATGGCTTTCCCTCGAATAAATAACATAAGTTTCTGGCTCCTGCCCCCCTCATTCCTTCTTCTACTAGCCTCTTCTGGCGTAGAGGCTGGGGTTGGTACGGGATGAACCGTCTACCCCCCGCTAGCCGGCAACCTTGCCCATGCTGGAGCCTCCGTAGACCTAGCCATTTTCTCTCTTCACCTGGCCGGAGTCTCCTCCATCCTTGGTTCAATCAACTTTATTACCACAATTATCAACATAAAACCCCCAGCAATTTCTCAATATCAAACCCCATTATTCATTTGAGCCCTGCTAGTTACTACTGTGCTTCTGCTGCTGTCATTACCAGTTTTAGCTGCAGGAATTACGATGCTACTAACAGATCGAAACCTAAACACAACATTTTTTGACCCGGCAGGCGGGGGGGATCCAATCCTCTACCAACACTTATTC

>75B1_Mormyrops_anguilloides Mormyrops anguilloides

GCACTGAGCCTCCTTATCCGAGCGGAACTAAACCAACCAGGGGCCCTGCTTGGCGACGACCAGATTTATAATGTTATCGTTACAGCACACGCCTTCGTAATAATTTTCTTCATAGTAATGCCAATCATGATCGGCGGTTTTGGCAACTGATTAATCCCCCTCATGCTCGGCGCCCCAGATATAGCATTTCCCCGAATGAACAACATAAGCTTTTGACTCCTACCACCATCCTTCCTCCTTCTACTTGCCTCTTCTGGAGTCGAAGCTGGAGTTGGGACAGGATGAACTGTCTACCCGCCCCTGGCGGGCAATTTAGCCCATGCCGGAGCCTCTGTAGACCTGGCCATTTTCTCCCTCCACTTAGCCGGTGTGTCCTCTATCCTTGGCTCTATCAACTTCATTACCACAATCATCAACATAAAACCCCCAGCAATCTCCCAATACCAAACCCCCCTTTTTATTTGAGCCCTATTAGTGACCACCGTCTTACTACTACTATCCCTGCCAGTCCTAGCTGCAGGAATCACAATACTACTAACAGATCGGAACCTGAATACAACATTCTTCGACCCGGCAGGCGGAGGAGACCCAATCCTCTACCAACACCTATTC

>96I7_Mormyrus_cf_tapirus

GGCACTGCACTGAGCCTCCTAATTCGAGCCGAACTAAACCAACCTGGGGCACTGCTTGGTGACGACCAGATTTATAATGTCATCGTCACAGCACACGCCTTCGTAATAATTTTCTTCATGGTAATACCAATTATAATTGGCGGATTCGGCAACTGACTAATTCCTCTCATGCTGGGCGCCCCCGACATGGCATTCCCCCGAATAAACAACATAAGCTTCTGACTCCTGCCCCCATCATTCCTGCTCTTGCTCGCCTCCTCTGGCGTGGAAGCCGGGGTCGGAACCGGATGAACTGTCTACCCACCCCTGGCCGGTAACCTGGCCCACGCAGGGGCCTCCGTAGACCTGGCTATTTTCTCCCTCCACCTAGCCGGAGTATCATCTATCCTAGGCTCAATCAACTTCATCACCACAATTATCAATATGAAACCACCAGCGATCTCCCAATACCAGACCCCTCTCTTCATCTGGGCTACTTTAGTAACCACAGTCCTGCTACTGCTATCGCTGCCAGTCCTCGCCGCAGGAATTACAATGCTGCTAACGGACCGAAACCTAAACACAACATTCTTTGACCCAGCAGGGGGAGGAGACCCAATCCTCTACCAACACCTATTCTGATTCTTTGGCC

>97A7_Mormyrops_caballus

GGCACTGCACTAAGCCTCCTTATCCGAGCGGAACTAAACCAACCGGGGGCCCTGCTTGGCGACGACCAGATTTATAATGTTATCGTCACAGCACACGCCTTCGTAATAATTTTCTTCATAGTAATGCCAATCATGATCGGCGGTTTTGGCAACTGATTAATCCCCCTCATGCTCGGCGCCCCAGATATAGCATTTCCCCGAATGAACAACATAAGCTTTTGACTCCTACCACCATCCTTCCTCCTTCTACTTGCCTCCTCTGGAGTCGAAGCTGGAGTTGGGACAGGATGAACTGTCTACCCGCCCCTGGCGGGCAATTTAGCCCATGCCGGAGCCTCTGTAGACCTGGCTATTTTCTCCCTCCACTTAGCCGGTGTATCCTCCATCCTTGGCTCTATCAACTTCATCACCACAATCATCAACATAAAACCCCCAGCAATCTCCCAATACCAAACCCCCCTTTTTATTTGAGCCCTATTAGTGACCACCGTCTTACTACTACTATCCCTGCCAGTCCTAGCTGCAGGAATCACAATACTACTAACAGATCGGAACCTGAATACAACATTCTTCGACCCAGCAGGCGGAGGAGACCCAATCCTCTACCAACACCTATTCTGATTCTTTGGCC

>97C3_Petrocephalus_christyi

GGCACTGCACTGAGCCTCCTGATCCGAGCAGAACTAAACCAACCTGGGGCCTTGCTTGGCGACGACCAGATTTATAATGTCATCGTTACTGCACATGCCTTCGTAATAATTTTCTTCATGGTAATACCAATTATAATCGGAGGGTTTGGCAACTGACTAATTCCACTCATGCTAGGCGCCCCAGACATGGCGTTCCCCCGAATAAATAACATAAGCTTCTGACTACTACCCCCATCCTTCCTCCTTCTCCTTGCTTCTTCAGGAGTAGAGGCCGGGGTTGGAACCGGCTGAACGGTCTACCCACCATTGGCCAGTAATTTAGCCCATGCCGGGGCCTCTGTTGACCTAGCCATCTTTTCTCTTCACCTGGCAGGAGTGTCCTCTATTTTAGGCTCAATCAACTTCATCACCACAATTATTAATATAAAACCACCAGCAATTTCCCAATACCAAACCCCACTATTCATCTGGTCCCTGCTAGTAACCACAGTACTACTCCTACTTTCCCTACCAGTCCTGGCTGCAGGCATTACTATATTATTAACAGACCGAAACCTAAACACAACATTCTTCGACCCAGCCGGAGGGGGAGACCCAATTCTATATCAACACCTCTTCTGATTCTTTGGCC

>97E3_Campylomormyrus_phantasticus

ACTGCACTRAGCCTACTSATCCRAGCGRAKYTAAACMAAMMTGGRRCSMTRCKTGKGGASGAYCAKATYTAYMAYKKYMYMGTYACMKCACRMKCMTYCKDAATRATTYTYTKVMTGGYMATRMCCMTWATGATCGGTGGCTTCGGCAACTGACTAATCCCCCTTATGCTCGGCGCCCCTGACATAGCMTTTCCYCGAATGAATAACATAAGYTTCTGRCTCCTGCCCCCATCRTTCCTTCTTCTRCTAGCCTCTTCTGGGGTAGAAGCTGGAGTTGGMACAGGGTGAACCGTYTATCCACCACTAGCCGGCAACCTTGCCCATGCTGGAGCCTCCGTAGACCTGGCTATTTTCTCCCTCCAYCTGGCYGGAGTCTCATCTATCCTGGGCTCAATYAACTTCATTACCACAATTATCAAYATAAAACCCCCAGCAATCTCCCAATACCAAACCCCAYTATTTATTTGAGCCCTGCTAGTAACTACCGTGCTYCTACTGCTATCAYTACCAGTTTTAGCTGCAGGAATTACTATGCTACTAACAGATCGAAACCTAAACACAACATTCTTTGACCCGGCAGGCGGGGGAGACCCGATCCTCTACCAACACTTGTTCTGATTCTTTGGCC

>97E4_Campylomormyrus_phantasticus

ACTGCACTGAGCCTACTSATCCRAGCGRAAYTAAACCAACCTGGGGCCMTGCKTGKAGASGACCAKATYTAYMAYGKYMYMSTYACMKCACRMKSMTTCTYAATRATTYTYTKVAYRSYMATRMCYMTWATGATCGGTGGCTTCGGCAACTGACTAATCCCCCTTATGCTCGGCGCCCCTGACATAGCATTTCCTCGAATGAATAACATAAGTTTCTGRCTCCTGCCCCCATCGTTCCTTCTTCTACTAGCCTCTTCTGGGGTAGAAGCTGGAGTTGGCACAGGGTGAACCGTTTATCCACCACTAGCCGGCAACCTTGCCCATGCTGGAGCCTCCGTAGACCTGGCTATTTTCTCCCTCCATCTGGCCGGAGTCTCATCTATCCTGGGCTCAATTAACTTCATTACCACAATTATCAACATAAAACCCCCAGCAATCTCCCAATACCAAACCCCATTATTTATTTGAGCCCTGCTAGTAACCACCGTGCTTCTACTGCTATCATTACCAGTTTTAGCTGCAGGAATTACTATGCTACTAACAGATCGAAACCTAAACACAACATTCTTTGACCCGGCAGGCGGGGGAGACCCGATCCTCTACCAACACTTGTTCTGATTCTTTGGCC

>100H3_paramormyrops

GGCACTGCACTGAGCCTTCTTATCCGAGCGGAACTAAACCAACCTGGAGCCCTACTTGGGGATGACCAGATTTATAATGTTATCGTCACAGCACACGCCTTCGTAATAATTTTCTTTATGGTAATGCCAATCATGATCGGCGGATTTGGCAACTGATTAATTCCACTTATGCTCGGCGCCCCTGACATGGCATTCCCCCGAATGAATAACATAAGCTTCTGGCTTCTACCCCCATCATTCCTTCTCCTACTCGCCTCTTCTGGTGTTGAAGCTGGAGTTGGGACAGGGTGAACCGTCTACCCACCACTAGCCGGCAATCTAGCCCATGCTGGAGCCTCCGTAGACCTGGCCATCTTCTCCCTCCATTTAGCCGGAGTCTCATCCATCCTTGGCTCAATTAACTTTATCACCACCATTATTAATATAAAACCCCCAGCAATTTCCCAGTACCAAACCCCACTTTTTATCTGAGCCCTGCTAGTAACCACTGTACTTCTACTACTCTCCTTACCAGTTTTAGCTGCAGGAATTACAATACTACTAACAGACCGAAACCTGAATACAACATTCTTTGACCCAGCTGGCGGAGGGGACCCAATCCTATACCAACACCTGTTCTGATTCTTTGGCAC

>100H8_paramormyrops

GGCACTGCACTGAGCCTTCTTATCCGAGCGGAACTAAACCAACCTGGAGCCCTACTTGGGGATGACCAGATTTATAATGTTATCGTCACAGCACACGCCTTCGTAATAATTTTCTTTATGGTAATGCCAATCATGATCGGCGGATTTGGCAACTGATTAATTCCACTTATGCTCGGCGCCCCTGACATGGCATTCCCCCGAATGAATAACATAAGCTTCTGGCTTCTACCCCCATCATTCCTTCTCCTACTCGCCTCTTCTGGTGTTGAAGCTGGAGTTGGGACAGGGTGAACCGTCTACCCACCACTAGCCGGCAATCTAGCCCATGCTGGAGCCTCCGTAGACCTGGCCATCTTCTCCCTCCATTTAGCCGGAGTCTCATCCATCCTTGGCTCAATTAACTTTATCACCACCATTATTAATATAAAACCCCCAGCAATTTCCCAGTACCAAACCCCACTTTTTATCTGAGCCCTGCTAGTAACCACTGTACTTCTACTACTCTCCTTACCAGTTTTAGCTGCAGGAATTACAATACTACTAACAGACCGAAACCTGAATACAACATTCTTTGACCCAGCTGGCGGAGGGGACCCAATCCTATACCAACACCTGTTCTGATTCTTTGGCAC
