# Supplementary material for: Trophic ecology of the African riverine elephant fishes (Mormyridae)
Source: Ecol Evol. 2024 Aug 27;14(8):e70173. doi: 10.1002/ece3.70173 (PMC11349487; doi:10.1002/ece3.70173)
Supplement: Supplementary file 2 — Table S1. Table S2. Table S3. [file ECE3-14-e70173-s001.pdf]

**code**	***species***	**d15N**	**d13C**	**Season**	**date**	**month**	**year**	**location**
**62D3**	*Campylomormyrus phantasticus*	12.236	-23.124	dry	8	March	2017	Sanaga, Nachtigal falls
**62D9**	*Campylomormyrus phantasticus*	12.253	-22.924	dry	8	March	2017	Sanaga, Nachtigal falls
**62F6**	*Campylomormyrus phantasticus*	12.016	-22.753	dry	10	March	2017	Sanaga, Nachtigal falls
**62C2**	*Campylomormyrus phantasticus*	12.262	-22.922	dry	8	March	2017	Sanaga, Nachtigal falls
**16338**	*Campylomormyrus phantasticus*	12.505	-23.715	wet	1	November	2018	Sanaga, Nachtigal falls
**16339**	*Campylomormyrus phantasticus*	11.942	-23.265	wet	1	November	2018	Sanaga, Nachtigal falls
**62G1**	*Campylomormyrus phantasticus*	12.278	-23.495	dry	11	March	2017	Sanaga, Nachtigal falls
**101B5**	*Campylomormyrus phantasticus*	12.619	-24.207	dry	19	February	2018	Sanaga, Nachtigal falls
**101D7**	*Campylomormyrus phantasticus*	12.430	-23.917	dry	19	February	2018	Sanaga, Nachtigal falls
**101E2**	*Campylomormyrus phantasticus*	12.195	-23.924	dry	20	February	2018	Sanaga, Nachtigal falls, upstream
**101E3**	*Campylomormyrus phantasticus*	12.017	-25.436	dry	20	February	2018	Sanaga, Nachtigal falls, upstream
**101E4**	*Campylomormyrus phantasticus*	12.333	-23.535	dry	20	February	2018	Sanaga, Nachtigal falls, upstream
**101G6**	*Campylomormyrus phantasticus*	12.170	-24.302	dry	21	February	2018	Avo'o
**15503**	*Campylomormyrus phantasticus*	12.040	-22.252	transition	11	July	2018	Sanaga
**15512**	*Campylomormyrus phantasticus*	12.184	-22.964	transition	11	July	2018	Sanaga
**15519**	*Campylomormyrus phantasticus*	12.141	-22.568	transition	11	July	2018	Sanaga
**15520**	*Campylomormyrus phantasticus*	12.229	-22.132	transition	11	July	2018	Sanaga
**15558**	*Campylomormyrus phantasticus*	12.261	-23.389	wet	5	September	2018	Sanaga, Nachtigal falls
**15601**	*Campylomormyrus phantasticus*	12.428	-23.519	wet	5	September	2018	Sanaga, Nachtigal falls
**14345**	*Campylomormyrus phantasticus*	12.139	-23.152	dry	21	March	2018	Sanaga, Nachtigal falls, upstream
**14363**	*Campylomormyrus phantasticus*	12.138	-24.563	dry	21	March	2018	Sanaga, Nachtigal falls, upstream
**14364**	*Campylomormyrus phantasticus*	12.092	-24.557	dry	21	March	2018	Sanaga, Nachtigal falls, upstream
**101G8**	*Campylomormyrus phantasticus*	12.059	-24.449	dry	21	February	2018	Avo'o
**14183**	*Campylomormyrus phantasticus*	12.184	-24.157	dry	20	February	2018	Sanaga, Nachtigal falls, upstream
**14184**	*Campylomormyrus phantasticus*	12.164	-23.947	dry	20	February	2018	Sanaga, Nachtigal falls, upstream
**14401**	*Campylomormyrus phantasticus*	12.161	-23.899	dry	22	March	2018	Sanaga, Nachtigal falls, upstream

**code**	***species***	**d15N**	**d13C**	**Season**	**date**	**month**	**year**	**location**
**15711**	*Campylomormyrus phantasticus*	12.126	-24.339	wet	8	September	2018	Sanaga, Nachtigal falls, upstream
**15712**	*Campylomormyrus phantasticus*	12.150	-23.381	wet	8	September	2018	Sanaga, Nachtigal falls, upstream
**15713**	*Campylomormyrus phantasticus*	12.189	-23.386	wet	8	September	2018	Sanaga, Nachtigal falls, upstream
**62A2**	*Campylomormyrus phantasticus*	11.871	-22.532	dry	7	March	2017	Sanaga, Nachtigal falls
**62C5**	*Campylomormyrus phantasticus*	11.779	-22.193	dry	8	March	2017	Sanaga, Nachtigal falls
**62C6**	*Campylomormyrus phantasticus*	11.999	-22.887	dry	8	March	2017	Sanaga, Nachtigal falls
**62D7**	*Hippopotamyrus castor*	11.335	-23.877	dry	9	March	2017	Sanaga, Nachtigal falls
**62A4**	*Hippopotamyrus castor*	12.259	-23.487	dry	7	March	2017	Sanaga, Nachtigal falls
**62A5**	*Hippopotamyrus castor*	12.055	-23.541	dry	7	March	2017	Sanaga, Nachtigal falls
**101D5**	*Hippopotamyrus castor*	12.610	-24.161	dry	19	February	2018	Sanaga, Nachtigal falls
**101D9**	*Hippopotamyrus castor*	12.165	-24.302	dry	20	February	2018	Sanaga, Nachtigal falls, upstream
**16340**	*Hippopotamyrus castor*	12.755	-24.085	wet	1	November	2018	Sanaga, Nachtigal falls
**16341**	*Hippopotamyrus castor*	12.315	-23.269	wet	1	November	2018	Sanaga, Nachtigal falls
**101E1**	*Hippopotamyrus castor*	11.561	-19.593	dry	20	February	2018	Sanaga, Nachtigal falls,upstream
**101G5**	*Hippopotamyrus castor*	12.444	-24.063	dry	21	February	2018	Avo'o
**15526**	*Hippopotamyrus castor*	11.485	-24.020	transition	11	July	2018	Sanaga, Nachtigal falls
**13582**	*Hippopotamyrus castor*	10.367	-28.910	dry	12	February	2018	Stream park (Djim river)
**15071**	*Hippopotamyrus castor*	11.183	-29.208	transition	29	May	2018	Stream park
**12402**	*Hippopotamyrus castor*	11.635	-23.155	wet	8	November	2017	Stream park (Mey river)
**14344**	*Hippopotamyrus castor*	11.497	-23.007	dry	21	March	2018	Sanaga, Nachtigal falls,upstream
**15667**	*Hippopotamyrus castor*	11.842	-21.219	wet	7	September	2018	Sanaga, Nachtigal falls,upstream
**15698**	*Hippopotamyrus castor*	11.820	-23.080	wet	7	September	2018	Sanaga, Nachtigal falls,upstream
**12464**	*Hippopotamyrus castor*	12.484	-23.733	wet	9	November	2017	Sanaga, Nachtigal falls,upstream
**14189**	*Hippopotamyrus castor*	11.458	-19.511	dry	20	February	2018	Sanaga, Nachtigal falls,upstream
**12313**	*Marcusenius mento*	11.386	-25.798	wet	25	August	2017	Stream park (Mpem river)
**14819**	*Marcusenius mento*	10.313	-31.395	transition	25	May	2018	Stream park (Mpem river)
**14833**	*Marcusenius mento*	11.227	-29.672	transition	26	May	2018	Stream park (Mpem river)

**code**	***species***	**d15N**	**d13C**	**Season**	**date**	**month**	**year**	**location**
**14860**	*Marcusenius mento*	10.308	-30.384	transition	26	May	2018	Stream park (Mpem river)
**12078**	*Marcusenius sanagaensis*	12.381	-24.519	wet	16	September	2017	Stream san (Asamba river)
**12084**	*Marcusenius sanagaensis*	10.340	-27.333	wet	16	Oktober	2017	Stream san (Asamba river)
**14452**	*Marcusenius sanagaensis*	11.061	-27.501	dry	23	March	2018	Stream san (Asamba river)
**12137**	*Marcusenius sanagaensis*	10.433	-28.338	wet	17	August	2017	Stream san (Nia river)
**62F9**	*Marcusenius sanagaensis*	10.907	-16.514	dry	11	March	2017	Sanaga, Nachtigal falls
**62D4**	*Marcusenius sanagaensis*	11.922	-20.360	dry	8	March	2017	Sanaga, Nachtigal falls
**12085**	*Marcusenius sanagaensis*	10.327	-27.288	wet	16	August	2017	Stream san (Asamba river)
**12086**	*Marcusenius sanagaensis*	10.227	-26.837	wet	16	August	2017	Stream san (Asamba river)
**12469**	*Marcusenius sanagaensis*	10.168	-26.533	wet	10	November	2017	Stream san (Asamba river)
**12477**	*Marcusenius sanagaensis*	10.214	-27.633	wet	10	November	2017	Stream san (Asamba river)
**14497**	*Marcusenius sanagaensis*	10.523	-27.795	dry	23	March	2018	Stream san (Asamba river)
**14498**	*Marcusenius sanagaensis*	10.587	-27.683	dry	23	March	2018	Stream san (Asamba river)
**14499**	*Marcusenius sanagaensis*	10.876	-26.652	dry	23	March	2018	Stream san (Asamba river)
**15212**	*Marcusenius sanagaensis*	10.433	-28.107	transition	7	June	2018	Stream san (Asamba river)
**15216**	*Marcusenius sanagaensis*	10.145	-28.087	transition	7	June	2018	Stream san (Asamba river)
**15536**	*Marcusenius sanagaensis*	10.757	-27.092	transition	12	July	2018	Stream san (Asamba river)
**15788**	*Marcusenius sanagaensis*	10.503	-27.383	wet	9	September	2018	Stream san (Asamba river)
**12187**	*Marcusenius sanagaensis*	10.407	-27.290	wet	19	August	2017	Stream san (Banga river)
**14586**	*Marcusenius sanagaensis*	9.416	-23.757	dry	25	March	2018	Stream san (Banga river)
**14587**	*Marcusenius sanagaensis*	9.382	-30.131	dry	25	March	2018	Stream san (Banga river)
**13680**	*Marcusenius sanagaensis*	9.954	-28.176	dry	13	February	2018	Stream park (Djim river)
**13681**	*Marcusenius sanagaensis*	10.952	-27.767	dry	13	February	2018	Stream park (Djim river)
**13682**	*Marcusenius sanagaensis*	10.641	-29.134	dry	13	February	2018	Stream park (Djim river)
**13683**	*Marcusenius sanagaensis*	10.289	-27.392	dry	13	February	2018	Stream park (Djim river)
**13684**	*Marcusenius sanagaensis*	10.356	-26.553	dry	13	February	2018	Stream park (Djim river)
**15342**	*Marcusenius sanagaensis*	8.787	-16.720	transition	9	June	2018	Stream san (Mekono river)

**code**	***species***	**d15N**	**d13C**	**Season**	**date**	**month**	**year**	**location**
**15343**	*Marcusenius sanagaensis*	9.053	-16.505	transition	9	June	2018	Stream san (Mekono river)
**15344**	*Marcusenius sanagaensis*	9.023	-15.979	transition	9	June	2018	Stream san (Mekono river)
**15346**	*Marcusenius sanagaensis*	8.731	-17.838	transition	9	June	2018	Stream san (Mekono river)
**15070**	*Marcusenius sanagaensis*	10.605	-27.595	transition	29	May	2018	Stream park (Mey river)
**12594**	*Marcusenius sanagaensis*	10.706	-29.910	wet	21	November	2017	Stream park (Mpem river)
**12619**	*Marcusenius sanagaensis*	11.551	-26.974	wet	21	November	2017	Stream park (Mpem river)
**12620**	*Marcusenius sanagaensis*	11.201	-26.750	wet	21	November	2017	Stream park (Mpem river)
**14831**	*Marcusenius sanagaensis*	9.962	-27.503	transition	26	May	2018	Stream park (Mpem river)
**14832**	*Marcusenius sanagaensis*	10.906	-27.292	transition	26	May	2018	Stream park (Mpem river)
**12114**	*Marcusenius sanagaensis*	10.763	-25.985	wet	17	August	2017	Stream san (Nia river)
**12136**	*Marcusenius sanagaensis*	10.156	-27.251	wet	17	August	2017	Stream san (Nia river)
**14548**	*Marcusenius sanagaensis*	10.650	-28.024	dry	24	March	2018	Stream san (Nia river)
**15920**	*Marcusenius sanagaensis*	10.827	-25.661	wet	14	September	2018	Stream san (Nia river)
**15921**	*Marcusenius sanagaensis*	10.511	-26.193	wet	14	September	2018	Stream san (Nia river)
**12556**	*Marcusenius sanagaensis*	9.701	-27.417	wet	15	November	2017	Stream san (Seele river)
**14690**	*Marcusenius sanagaensis*	10.782	-29.751	dry	27	March	2018	Stream san (Sele river)
**16136**	*Marcusenius sanagaensis*	10.699	-27.806	wet	16	September	2018	Stream san (Tede river)
**62A8**	*Mormyrops anguilloides*	11.624	-24.941	dry	7	March	2017	Sanaga, Nachtigal falls
**62AB3**	*Mormyrops anguilloides*	12.918	-22.165	dry	7	March	2017	Sanaga, Nachtigal falls
**62B4**	*Mormyrops anguilloides*	12.253	-24.423	dry	7	March	2017	Sanaga, Nachtigal falls
**62C1**	*Mormyrops anguilloides*	12.985	-21.488	dry	7	March	2017	Sanaga, Nachtigal falls
**15219**	*Mormyrops anguilloides*	12.739	-26.394	transition	7	June	2018	Stream san (Asamba river)
**12035**	*Mormyrops anguilloides*	12.504	-23.730	wet	14	August	2017	Stream san (Avo’o river)
**13943**	*Mormyrops anguilloides*	12.556	-34.964	dry	18	February	2018	Sanaga, Nachtigal falls
**101A8**	*Mormyrops anguilloides*	12.163	-25.657	dry	18	February	2018	Sanaga, Nachtigal falls
**16267**	*Mormyrops anguilloides*	12.310	-23.132	wet	29	October	2018	Sanaga, Nachtigal falls
**16290**	*Mormyrops anguilloides*	13.024	-22.971	wet	30	October	2018	Sanaga, Nachtigal falls

**code**	***species***	**d15N**	**d13C**	**Season**	**date**	**month**	**year**	**location**
**16303**	*Mormyrops anguilloides*	12.114	-24.866	wet	31	October	2018	Sanaga, Nachtigal falls
**16304**	*Mormyrops anguilloides*	12.352	-24.324	wet	31	October	2018	Sanaga, Nachtigal falls
**16305**	*Mormyrops anguilloides*	11.134	-26.195	wet	31	October	2018	Sanaga, Nachtigal falls
**16311**	*Mormyrops anguilloides*	11.632	-28.465	wet	31	October	2018	Sanaga, Nachtigal falls
**16312**	*Mormyrops anguilloides*	10.914	-29.213	wet	31	October	2018	Sanaga, Nachtigal falls
**16313**	*Mormyrops anguilloides*	12.788	-23.492	wet	31	October	2018	Sanaga, Nachtigal falls
**16314**	*Mormyrops anguilloides*	12.622	-24.075	wet	1	November	2018	Stream san (Wala river)
**16326**	*Mormyrops anguilloides*	12.185	-22.219	wet	1	November	2018	Stream san (Wala river)
**16332**	*Mormyrops anguilloides*	11.019	-26.016	wet	1	November	2018	Sanaga, Nachtigal falls
**16333**	*Mormyrops anguilloides*	12.742	-23.729	wet	1	November	2018	Sanaga, Nachtigal falls
**101C6**	*Mormyrops anguilloides*	13.124	-24.278	dry	19	February	2018	Sanaga, Nachtigal falls
**13446**	*Mormyrops anguilloides*	11.308	-28.121	dry	12	February	2018	Stream park (Djim river)
**12408**	*Mormyrops anguilloides*	11.710	-26.588	wet	8	November	2017	Sanaga, Nachtigal falls,upstream
**14391**	*Mormyrops anguilloides*	11.508	-30.994	dry	21	March	2018	Sanaga, Nachtigal falls,upstream
**12105**	*Mormyrops anguilloides*	12.093	-25.032	wet	17	August	2017	Stream san (Nia river)
**12555**	*Mormyrops anguilloides*	11.726	-28.883	wet	15	November	2017	Stream san (Sele river)
**62D1**	*Mormyrops anguilloides*	12.730	-22.191	dry	8	March	2017	Sanaga, Nachtigal falls
**62D2**	*Mormyrops anguilloides*	12.090	-22.875	dry	8	March	2017	Sanaga, Nachtigal falls
**62E2**	*Mormyrops anguilloides*	11.943	-24.203	dry	9	March	2017	Sanaga, Nachtigal falls
**62F1**	*Mormyrops anguilloides*	11.792	-23.266	dry	9	March	2017	Sanaga, Nachtigal falls
**62G3**	*Mormyrops anguilloides*	11.753	-27.546	dry	11	March	2017	Sanaga, Nachtigal falls
**12034**	*Mormyrops caballus*	12.715	-22.931	wet	14	August	2017	Stream san (Avo'o river)
**16291**	*Mormyrops caballus*	12.873	-21.914	wet	30	October	2018	Sanaga, Nachtigal falls
**62F3**	*Mormyrops caballus*	12.328	-22.292	dry	10	March	2017	Asamba river
**62G4**	*Mormyrops caballus*	12.347	-22.700	dry	11	March	2017	Sanaga, Nachtigal falls
**101A9**	*Mormyrops caballus*	12.460	-22.254	dry	18	February	2018	Sanaga, Nachtigal falls
**16334**	*Mormyrops caballus*	12.489	-23.662	wet	1	November	2018	Sanaga, Nachtigal falls

code	*species*	d15N	d13C	Season	date	month	year	location
**16342**	*Mormyrops caballus*	12.126	-23.492	wet	2	November	2018	Stream san (Wala river)
**101B4**	*Mormyrops caballus*	12.302	-24.865	dry	19	February	2018	Sanaga, Nachtigal falls
**101C7**	*Mormyrops caballus*	12.483	-22.575	dry	19	February	2018	Sanaga, Nachtigal falls
**101C4**	*Mormyrops caballus*	11.689	-24.538	dry	19	February	2018	Sanaga, Nachtigal falls
**101F7**	*Mormyrops caballus*	12.555	-22.037	dry	21	February	2018	Avo'o
**101F8**	*Mormyrops caballus*	12.292	-22.219	dry	21	February	2018	Avo'o
**14005**	*Mormyrops caballus*	12.338	-22.291	dry	19	February	2018	Sanaga, Nachtigal falls
**14431**	*Mormyrops caballus*	12.635	-21.889	dry	22	March	2018	Sanaga, Nachtigal falls, upstream
**14443**	*Mormyrops caballus*	12.453	-22.631	dry	22	March	2018	Sanaga, Nachtigal falls, upstream
**14675**	*Mormyrops caballus*	11.857	-29.457	dry	27	March	2018	Stream san (Sele river)
**14676**	*Mormyrops caballus*	11.890	-29.351	dry	27	March	2018	Stream san (Sele river)
**15981**	*Mormyrops caballus*	11.826	-30.549	wet	15	September	2018	Stream san (Sele river)
**14207**	*Mormyrops caballus*	12.460	-22.049	dry	21	February	2018	Sanaga, Nachtigal falls, upstream
**14218**	*Mormyrops caballus*	12.207	-22.233	dry	21	February	2018	Sanaga, Nachtigal falls, upstream
**62B8**	*Mormyrops caballus*	12.326	-23.362	dry	7	March	2017	Sanaga, Nachtigal falls
**62C8**	*Mormyrus tapirus*	12.150	-24.227	dry	8	March	2017	Sanaga, Nachtigal falls
**62C9**	*Mormyrus tapirus*	12.493	-24.722	dry	8	March	2017	Sanaga, Nachtigal falls
**62D5**	*Mormyrus tapirus*	11.352	-26.149	dry	8	March	2017	Sanaga, Nachtigal falls
**62D6**	*Mormyrus tapirus*	11.976	-23.856	dry	8	March	2017	Sanaga, Nachtigal falls
**62D8**	*Mormyrus tapirus*	9.520	-26.765	dry	9	March	2017	Sanaga, Nachtigal falls
**62E5**	*Mormyrus tapirus*	11.953	-24.133	dry	9	March	2017	Sanaga, Nachtigal falls
**62E6**	*Mormyrus tapirus*	12.107	-23.693	dry	9	March	2017	Sanaga, Nachtigal falls
**62E8**	*Mormyrus tapirus*	11.059	-24.568	dry	9	March	2017	Sanaga, Nachtigal falls
**62E7**	*Mormyrus tapirus*	11.638	-24.148	dry	9	March	2017	Sanaga, Nachtigal falls
**62A1**	*Mormyrus tapirus*	10.851	-24.324	dry	7	March	2017	Sanaga, Nachtigal falls
**62A9**	*Mormyrus tapirus*	10.830	-24.410	dry	7	March	2017	Sanaga, Nachtigal falls
**62B9**	*Mormyrus tapirus*	11.605	-24.940	dry	7	March	2017	Sanaga, Nachtigal falls

code	*species*	d15N	d13C	Season	date	month	year	location
**62C3**	*Mormyrus tapirus*	13.474	-25.555	dry	8	March	2017	Sanaga, Nachtigal falls
**62G2**	*Mormyrus tapirus*	11.871	-21.922	dry	11	March	2017	Sanaga, Nachtigal falls
**62G5**	*Mormyrus tapirus*	11.248	-26.525	dry	11	March	2017	Sanaga, Nachtigal falls
**101 E8**	*Mormyrus tapirus*	12.322	-23.204	dry	20	February	2018	Sanaga, Nachtigal falls,upstream
**101A7**	*Mormyrus tapirus*	12.447	-24.355	dry	18	February	2018	Sanaga, Nachtigal falls
**101B1**	*Mormyrus tapirus*	11.688	-24.368	dry	18	February	2018	Sanaga, Nachtigal falls
**101B2**	*Mormyrus tapirus*	11.385	-23.723	dry	18	February	2018	Sanaga, Nachtigal falls
**101B3**	*Mormyrus tapirus*	10.726	-27.153	dry	18	February	2018	Sanaga, Nachtigal falls
**101B6**	*Mormyrus tapirus*	11.576	-25.294	dry	19	February	2018	Sanaga, Nachtigal falls
**101B8**	*Mormyrus tapirus*	11.281	-25.500	dry	19	February	2018	Sanaga, Nachtigal falls
**101C1**	*Mormyrus tapirus*	12.833	-21.620	dry	19	February	2018	Sanaga, Nachtigal falls
**101B9**	*Mormyrus tapirus*	11.919	-21.148	dry	19	February	2018	Sanaga, Nachtigal falls
**101C3**	*Mormyrus tapirus*	12.175	-23.541	dry	19	February	2018	Sanaga, Nachtigal falls
**101C9**	*Mormyrus tapirus*	11.904	-24.412	dry	19	February	2018	Sanaga, Nachtigal falls
**16281**	*Mormyrus tapirus*	10.870	-23.804	wet	30	October	2018	Sanaga, Nachtigal falls
**16293**	*Mormyrus tapirus*	12.684	-23.020	wet	30	October	2018	Sanaga, Nachtigal falls
**15523**	*Mormyrus tapirus*	12.066	-23.650	transition	11	July	2018	Sanaga, Nachtigal falls, upstream
**15524**	*Mormyrus tapirus*	9.359	-28.020	transition	11	July	2018	Sanaga, Nachtigal falls, upstream
**14203**	*Mormyrus tapirus*	12.031	-24.422	dry	21	February	2018	Sanaga, Nachtigal falls, upstream
**14204**	*Mormyrus tapirus*	12.017	-22.717	dry	21	February	2018	Sanaga, Nachtigal falls, upstream
**14205**	*Mormyrus tapirus*	11.951	-20.869	dry	21	February	2018	Sanaga, Nachtigal falls, upstream
**12361**	*Mormyrus tapirus*	12.000	-22.953	wet	6	November	2017	Stream san (Wala river)
**14187**	*Mormyrus tapirus*	11.83	-24.065	dry	20	February	2018	Sanaga, Nachtigal falls, upstream
**14418**	*Mormyrus tapirus*	13.454	-22.491	dry	22	March	2018	Sanaga, Nachtigal falls, upstream
**16337**	*Mormyrus tapirus*	11.617	-22.912	wet	1	November	2018	Sanaga, Nchtigal falls
**16347**	*Mormyrus tapirus*	11.532	-22.833	wet	2	November	2018	Stream san (Wala river)
**16348**	*Mormyrus tapirus*	11.750	-21.858	wet	2	November	2018	Stream san (Wala river)

**code**	***species***	**d15N**	**d13C**	**Season**	**date**	**month**	**year**	**location**
**16315**	*Mormyrus tapirus*	11.763	-24.233	wet	1	November	2018	Stream san (Wala river)
**16321**	*Mormyrus tapirus*	11.671	-23.794	wet	1	November	2018	Stream san (Wala river)
**16323**	*Mormyrus tapirus*	12.198	-23.063	wet	1	November	2018	Stream san (Wala river)
**16324**	*Mormyrus tapirus*	12.753	-23.218	wet	1	November	2018	Stream san (Wala river)
**16335**	*Mormyrus tapirus*	12.224	-23.426	wet	1	November	2018	Sanaga, Nachtigal falls
**16336**	*Mormyrus tapirus*	12.289	-23.361	wet	1	November	2018	Sanaga, Nachtigal falls
**16346**	*Mormyrus tapirus*	11.865	-23.129	wet	2	November	2018	Stream san (Wala river)
**15037**	*Mormyrus tapirus*	10.902	-28.512	transition	29	May	2018	Stream park (Mpem river)
**15045**	*Mormyrus tapirus*	9.859	-30.128	transition	29	May	2018	Stream park (Mpem river)
**14825**	*Mormyrus tapirus*	9.888	-30.049	transition	26	May	2018	Stream park (Mpem river)
**14835**	*Mormyrus tapirus*	11.454	-27.864	transition	26	May	2018	Stream park (Mpem river)
**14346**	*Mormyrus tapirus*	11.305	-26.256	dry	21	March	2018	Sanaga, Nachtigal falls, upstream
**12360**	*Mormyrus tapirus*	11.775	-22.858	wet	6	November	2017	Stream san (Wala river)
**62A3**	*Mormyrus* sp. "long snout"	11.490	-23.122	dry	7	March	2017	Sanaga, Nachtigal falls
**62A6**	*Mormyrus* sp. "long snout"	11.443	-23.367	dry	7	March	2017	Sanaga, Nachtigal falls
**62A7**	*Mormyrus* sp. "long snout"	11.329	-22.697	dry	7	March	2017	Sanaga, Nachtigal falls
**12160**	*Mormyrus* sp. "short snout"	12.558	-20.070	wet	18	August	2017	Sanaga, Nachtigal falls, upstream
**12445**	*Mormyrus* sp. "short snout"	12.963	-21.937	wet	9	November	2017	Sanaga, Nachtigal falls, upstream
**12446**	*Mormyrus* sp. "short snout"	12.431	-20.701	wet	9	November	2017	Sanaga, Nachtigal falls, upstream
**14181**	*Mormyrus* sp. "short snout"	12.673	-22.421	dry	20	February	2018	Sanaga, Nachtigal falls, upstream
**14185**	*Mormyrus* sp. "short snout"	12.326	-20.721	dry	20	February	2018	Sanaga, Nachtigal falls, upstream
**14206**	*Mormyrus* sp. "short snout"	11.981	-22.450	dry	21	February	2018	Sanaga, Nachtigal falls, upstream
**15730**	*Mormyrus* sp. "short snout"	12.435	-21.635	wet	8	September	2018	Sanaga, Nachtigal falls, upstream
**15734**	*Mormyrus* sp. "short snout"	12.599	-20.167	wet	8	September	2018	Sanaga, Nachtigal falls, upstream
**15699**	*Mormyrus* sp. "short snout"	12.134	-19.847	wet	7	September	2018	Sanaga, Nachtigal falls, upstream
**62C7**	*Mormyrus* sp. "short snout"	11.750	-20.700	dry	7	March	2017	Sanaga, Nachtigal falls
**62B1**	*Mormyrus* sp. "short snout"	11.960	-20.014	dry	7	March	2017	Sanaga, Nachtigal falls

**code**	***species***	**d15N**	**d13C**	**Season**	**date**	**month**	**year**	**location**
**62B2**	*Mormyrus* sp. "short snout"	12.097	-18.485	dry	7	March	2017	Sanaga, Nachtigal falls
**62B5**	*Mormyrus* sp. "short snout"	11.875	-20.895	dry	7	March	2017	Sanaga, Nachtigal falls
**62B6**	*Mormyrus* sp. "short snout"	12.021	-22.138	dry	7	March	2017	Sanaga, Nachtigal falls
**62B7**	*Mormyrus* sp. "short snout"	12.458	-21.862	dry	7	March	2017	Sanaga, Nachtigal falls
**62C4**	*Mormyrus* sp. "short snout"	12.490	-22.204	dry	8	March	2017	Sanaga, Nachtigal falls
**62F8**	*Mormyrus* sp. "short snout"	12.163	-22.555	dry	11	March	2017	Sanaga, Nachtigal falls
**101A6**	*Mormyrus* sp. "short snout"	11.876	-18.652	dry	18	February	2018	Sanaga, Nachtigal falls
**101B7**	*Mormyrus* sp. "short snout"	12.638	-19.068	dry	19	February	2018	Sanaga, Nachtigal falls
**101C2**	*Mormyrus* sp. "short snout"	12.427	-19.405	dry	19	February	2018	Sanaga, Nachtigal falls
**101D2**	*Mormyrus* sp. "short snout"	11.740	-18.305	dry	19	February	2018	Sanaga, Nachtigal falls
**101D3**	*Mormyrus* sp. "short snout"	12.007	-21.204	dry	19	February	2018	Sanaga, Nachtigal falls
**101D4**	*Mormyrus* sp. "short snout"	11.833	-19.744	dry	19	February	2018	Sanaga, Nachtigal falls
**101D6**	*Mormyrus* sp. "short snout"	12.746	-21.916	dry	19	February	2018	Sanaga, Nachtigal falls
**101 E9**	*Mormyrus* sp. "short snout"	12.504	-19.260	dry	20	February	2018	Sanaga, Nachtigal falls, upstream
**101F1**	*Mormyrus* sp. "short snout"	12.265	-20.308	dry	20	February	2018	Sanaga, Nachtigal falls, upstream
**101F4**	*Mormyrus* sp. "short snout"	12.266	-21.895	dry	20	February	2018	Sanaga, Nachtigal falls, upstream
**101F5**	*Mormyrus* sp. "short snout"	12.187	-22.424	dry	20	February	2018	Sanaga, Nachtigal falls, upstream
**101F2**	*Mormyrus* sp. "short snout"	11.988	-18.628	dry	20	February	2018	Sanaga, Nachtigal falls, upstream
**101F3**	*Mormyrus* sp. "short snout"	11.806	-19.274	dry	20	February	2018	Sanaga, Nachtigal falls, upstream
**101F9**	*Mormyrus* sp. "short snout"	12.547	-21.291	dry	21	February	2018	Avo'o
**101G1**	*Mormyrus* sp. "short snout"	12.188	-21.100	dry	21	February	2018	Avo'o
**101G3**	*Mormyrus* sp. "short snout"	12.054	-20.109	dry	21	February	2018	Avo'o
**101G4**	*Mormyrus* sp. "short snout"	12.567	-21.197	dry	21	February	2018	Avo'o
**101G2**	*Mormyrus* sp. "short snout"	12.295	-23.053	dry	21	February	2018	Avo'o
**13470**	*Paramormyrops batesii*	11.351	-26.533	dry	12	February	2018	Stream park (Djim river)
**13679**	*Paramormyrops batesii*	9.927	-28.046	dry	13	February	2018	Stream park (Djim river)
**code**	***species***	**d15N**	**d13C**	**Season**	**date**	**month**	**year**	**location**

**13023**	*Paramormyrops batesii*	11.049	-28.974	dry	6	February	2018	Stream park (Mey river)
**12649**	*Paramormyrops batesii*	10.338	-27.146	wet	22	November	2017	Stream park (Mey river)
**15881**	*Petrocephalus christyi*	12.546	-22.733	wet	14	September	2018	Stream san (Nia river)
**62E1**	*Petrocephalus christyi*	11.435	-22.647	dry	9	March	2017	Sanaga, Nachtigal falls
**62G6**	*Petrocephalus christyi*	12.168	-22.199	dry	11	March	2017	Sanaga, Nachtigal falls
**62E4**	*Petrocephalus christyi*	12.263	-21.881	dry	9	March	2017	Sanaga, Nachtigal falls
**62F7**	*Petrocephalus christyi*	12.132	-21.645	dry	10	March	2017	Sanaga, Nachtigal falls
**14453**	*Petrocephalus christyi*	10.978	-32.835	dry	23	March	2018	Stream san (Asamba river)
**14455**	*Petrocephalus christyi*	11.530	-27.179	dry	23	March	2018	Stream san (Asamba river)
**14456**	*Petrocephalus christyi*	11.577	-27.098	dry	23	March	2018	Stream san (Asamba river)
**14457**	*Petrocephalus christyi*	10.257	-26.428	dry	23	March	2018	Stream san (Asamba river)
**15542**	*Petrocephalus christyi*	11.877	-28.995	transition	12	July	2018	Stream san (Asamba river)
**13828**	*Petrocephalus christyi*	11.321	-33.540	dry	15	February	2018	Stream park (Djim river)
**13829**	*Petrocephalus christyi*	11.324	-30.368	dry	15	February	2018	Stream park (Djim river)
**13830**	*Petrocephalus christyi*	10.295	-36.892	dry	15	February	2018	Stream park (Djim river)
**13831**	*Petrocephalus christyi*	10.766	-34.844	dry	15	February	2018	Stream park (Djim river)
**13832**	*Petrocephalus christyi*	11.352	-35.719	dry	15	February	2018	Stream park (Djim river)
**12827**	*Petrocephalus christyi*	12.111	-29.267	dry	5	February	2018	Stream park (Mpem river)
**12828**	*Petrocephalus christyi*	11.278	-33.720	dry	5	February	2018	Stream park (Mpem river)
**12829**	*Petrocephalus christyi*	12.388	-27.759	dry	5	February	2018	Stream park (Mpem river)
**14192**	*Petrocephalus christyi*	12.459	-23.751	dry	20	February	2018	Sanaga, Nachtigal falls, upstream
**14543**	*Petrocephalus christyi*	11.223	-29.351	dry	24	March	2018	Stream san (Nia river)
**14544**	*Petrocephalus christyi*	11.563	-28.448	dry	24	March	2018	Stream san (Nia river)
**14545**	*Petrocephalus christyi*	11.299	-26.535	dry	24	March	2018	Stream san (Nia river)
**14679**	*Petrocephalus christyi*	12.970	-29.194	dry	27	March	2018	Stream san (Sele river)
**15416**	*Petrocephalus christyi*	13.152	-27.259	transition	10	June	2018	Stream san (Sele river)
**15417**	*Petrocephalus christyi*	12.439	-26.535	transition	10	June	2018	Stream san (Sele river)
**code**	***species***	**d15N**	**d13C**	**Season**	**date**	**month**	**year**	**location**

**14767**	*Petrocephalus christyi*	11.380	-26.880	dry	28	March	2018	Stream san (Tede river)
**14768**	*Petrocephalus christyi*	12.288	-32.244	dry	28	March	2018	Stream san (Tede river)
**14769**	*Petrocephalus christyi*	11.897	-28.535	dry	28	March	2018	Stream san (Tede river)
**14770**	*Petrocephalus christyi*	11.899	-24.655	dry	28	March	2018	Stream san (Tede river)
**15482**	*Petrocephalus christyi*	12.509	-26.325	transition	11	June	2018	Stream san (Tede river)
**15483**	*Petrocephalus christyi*	12.435	-26.295	transition	11	June	2018	Stream san (Tede river)
**15485**	*Petrocephalus christyi*	12.115	-29.487	transition	11	June	2018	Stream san (Tede river)
**15486**	*Petrocephalus christyi*	11.962	-33.244	transition	11	June	2018	Stream san (Tede river)
**15487**	*Petrocephalus christyi*	12.452	-27.421	transition	11	June	2018	Stream san (Tede river)
**101A1**	*Petrocephalus christyi*	11.378	-24.642	dry	18	February	2018	Sanaga, Nachtigal falls
**101A2**	*Petrocephalus christyi*	11.366	-24.473	dry	18	February	2018	Sanaga, Nachtigal falls
**101A3**	*Petrocephalus christyi*	12.837	-23.437	dry	18	February	2018	Sanaga, Nachtigal falls
**101A5**	*Petrocephalus christyi*	11.519	-24.078	dry	18	February	2018	Sanaga, Nachtigal falls
**101A4**	*Petrocephalus christyi*	12.471	-23.293	dry	18	February	2018	Sanaga, Nachtigal falls
**101C5**	*Petrocephalus christyi*	11.607	-26.353	dry	19	February	2018	Sanaga, Nachtigal falls
**101D1**	*Petrocephalus christyi*	12.449	-23.877	dry	19	February	2018	Sanaga, Nachtigal falls
**101 E5**	*Petrocephalus christyi*	12.126	-23.541	dry	20	February	2018	Sanaga, Nachtigal falls, upstream
**101 E6**	*Petrocephalus christyi*	12.089	-21.085	dry	20	February	2018	Sanaga, Nachtigal falls, upstream
**101 E7**	*Petrocephalus christyi*	12.476	-21.758	dry	20	February	2018	Sanaga, Nachtigal falls, upstream

Table S2: sample list overview trophic samples

sample	group	Amount	d15N	d13C	N%	C%
benthic	amphipoda	0.49	7.26	-24.98	10.00%	37.50%
benthic	amphipoda	0.512	7.10	-24.96	6.89%	31.57%
benthic	anisoptera	0.536	6.99	-27.05	11.54%	46.08%
benthic	anisoptera	0.542	6.49	-22.04	11.11%	43.57%
stomach content	anisoptera	0.415	5.80	-22.69	9.03%	50.73%
stomach content	coleoptera	0.522	7.20	-30.58	10.77%	48.42%
stomach content	coleoptera	0.465	6.20	-26.07	10.67%	47.15%
benthic	coleoptera Hyrdophillidae	0.532	5.81	-21.12	11.74%	47.83%
stomach content	coleoptera larvae Elmidae	0.557	5.37	-27.33	9.83%	46.10%
stomach content	coleoptera larvae Elmidae	0.506	5.47	-24.90	9.80%	46.29%
benthic	coleoptera larvae Elmidae	0.488	3.94	-19.96	9.66%	46.44%
stomach content	coleoptera	0.5	6.35	-26.90	9.87%	48.49%
stomach content	diptera	0.337	6.84	-26.24	10.95%	45.08%
stomach content	diptera	0.321	6.99	-21.23	9.77%	42.51%
stomach content	diptera	0.535	7.32	-16.68	11.39%	44.38%
stomach content	ephemeroptera	0.283	6.56	-24.49	9.58%	38.97%
stomach content	gastropoda	0.52	5.94	-17.82	1.99%	7.12%
benthic	gastropoda	0.489	6.77	-20.03	2.92%	21.55%
benthic	gastropoda	0.47	6.56	-18.06	1.54%	16.16%
benthic	gastropoda	0.516	6.23	-14.77	1.05%	14.77%
benthic	hemiptera	0.536	7.92	-25.19	11.70%	47.75%
stomach content	lepidoptera	0.461	6.15	-24.04	8.49%	42.58%
stomach content	lepidoptera	0.471	6.70	-23.89	9.34%	45.27%
stomach content	lepidoptera	0.452	7.31	-21.60	9.59%	48.76%
stomach content	lepidoptera pupa	0.489	6.15	-20.07	9.06%	44.73%
benthic	odonata	0.545	6.72	-22.73	11.52%	44.11%
benthic	odonata	0.468	7.26	-21.53	12.08%	43.81%
benthic	plant	2.528	7.55	-22.99	4.02%	36.91%
benthic	plant	2.986	8.47	-20.39	1.77%	37.16%
stomach content	plecoptera	0.31	4.83	-25.74	9.12%	43.90%
stomach content	trichoptera	0.419	5.70	-26.08	7.01%	57.42%
benthic	zygoptera	0.462	7.77	-22.95	12.52%	44.42%
benthic	zygoptera	0.532	7.78	-22.80	11.45%	44.05%

Table S3: results of comparison tests (single-factor ANOVAs and Kruskal-Wallis tests) for species with transition season samples testing the factor of locality

single-factor ANOVA	p-value	df	Tukey HSD test	dry vs wet	dry vs transition	wet vs transition
Mormyrus tapirus δ13C without Mpem	0.033*	2		0.056 p adj. 0.133	0.117 p adj. 0.257	0.022* p adj. 0.057
Mormyrus tapirus δ15N without Mpem	0.107	2				
Marcusenius sanagaensis δ15N without Mekono	0.765	2				
Petrocephalus christyi δ15N dry vs transition without Mpem	0.011*	1				
**Kruskal-Wallis test**			Dunn's test with Bonferroni adjusted p-values			
Marcusenius sanagaensis δ15N without Mpem	0.045*	2		0.879	0.038**	0.321
Petrocephalus christyi δ13C dry vs transition without Mpem	0.199	1				

*p-value below 0.05, **adjusted p-value below 0.05
